# Supplementary figures and images for: Sequence/structural analysis of xylem proteome emphasizes pathogenesis-related proteins, chitinases and β-1, 3-glucanases as key players in grapevine defense against Xylella fastidiosa
Source: PeerJ. 2016 May 24;4:e2007. doi: 10.7717/peerj.2007 (PMC4888286; doi:10.7717/peerj.2007)

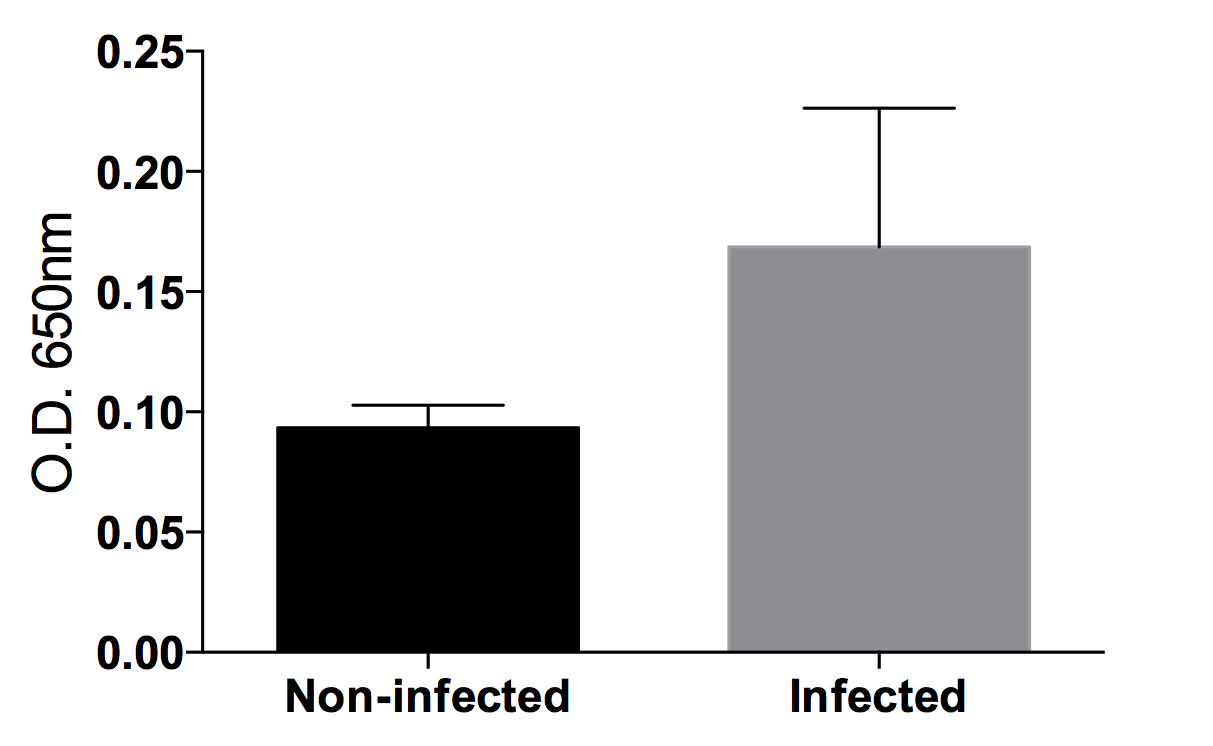

Supplement: Figure S1 — The anti-Xylella antibody (Agdia) is capable of detecting the dilute bacterial quantities in xylem sap, despite some background signal. [file peerj-04-2007-s001.png]

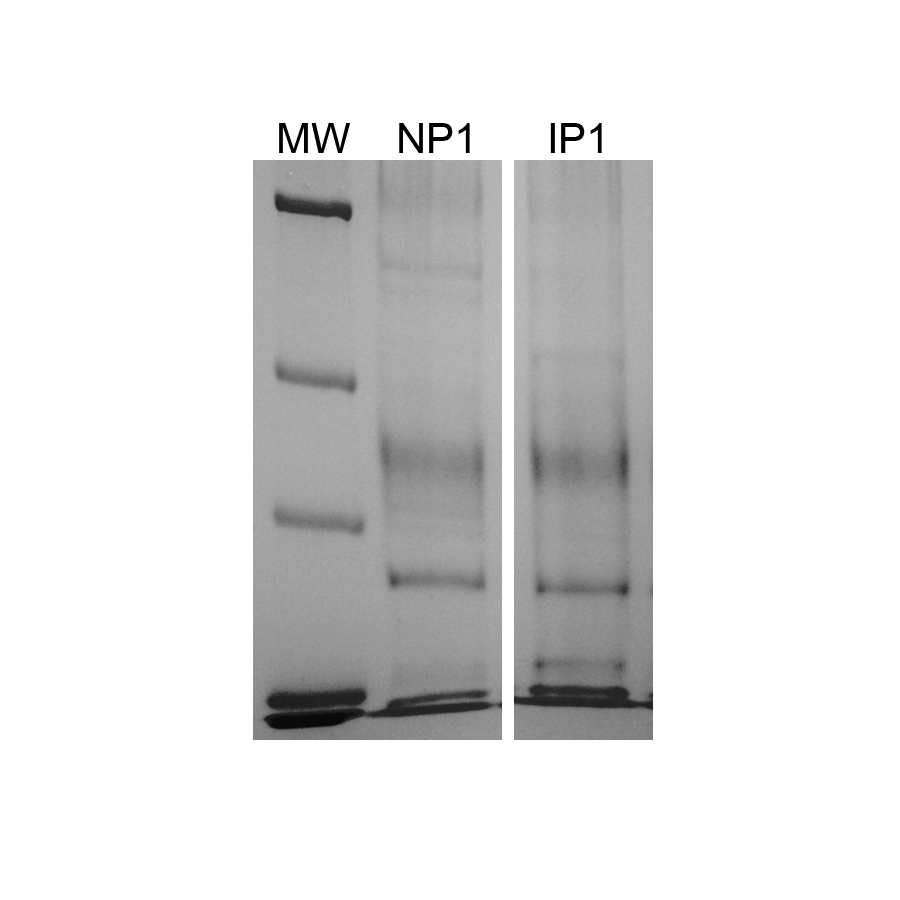

Supplement: Figure S2 — Imperial staining of SDS-PAGE with 5 µg representative xylem sap protein samples from non-infected (NP1) and infected (IP1) grapevines with Xylella fastidiosa used for mass spectrometry. [file peerj-04-2007-s002.png]
