# Supplementary material for: Sequence/structural analysis of xylem proteome emphasizes pathogenesis-related proteins, chitinases and β-1, 3-glucanases as key players in grapevine defense against Xylella fastidiosa
Source: PeerJ. 2016 May 24;4:e2007. doi: 10.7717/peerj.2007 (PMC4888286; doi:10.7717/peerj.2007)
Supplement: File S3 [file peerj-04-2007-s005.zip › perl_scripts/cgi-bin/frames.html]

Active Site Prediction and Protein Function Classification Chakraborty


## Active Site Prediction and Protein Function Classification

## Links

- Download source
- Database
- Web Server
- APBS Data
- BJR lab

# Catalytic Active Site Prediction (CLASP)

CLASP uses structural and electrostatic properties in a protein to predict its function. A motif from the protein of known function and 3D structure provides the initial template for searching spatially congruent matches (example motif ). These matches are then pruned by the observation that the potential difference (calculated using APBS) in pairs of residues in active sites are highly correlated. Our method can interrogate any given hypothetical protein with known structure for various motifs ( either derived from the CSA database or added by us) and rank the matches. This further allows CLASP in classifying promiscuous and moonlighting activities associated with the protein.

Some of the most promiscuous proteins as predicted by CLASP

Copyright © 2007. Company name all rights reserved
