# Supplementary material for: Sequence/structural analysis of xylem proteome emphasizes pathogenesis-related proteins, chitinases and β-1, 3-glucanases as key players in grapevine defense against Xylella fastidiosa
Source: PeerJ. 2016 May 24;4:e2007. doi: 10.7717/peerj.2007 (PMC4888286; doi:10.7717/peerj.2007)
Supplement: File S3 [file peerj-04-2007-s005.zip › perl_scripts/cgi-bin/KK.html]

Content-type: text/html

List of PDBs searched for elastase motif

| Column1 | Column2 | Column3 |
| --- | --- | --- |
| Red | Blue | Yellow |
| Cyan | Orange | Magenta |
| A wide row | | |
