# Supplementary material for: Sequence/structural analysis of xylem proteome emphasizes pathogenesis-related proteins, chitinases and β-1, 3-glucanases as key players in grapevine defense against Xylella fastidiosa
Source: PeerJ. 2016 May 24;4:e2007. doi: 10.7717/peerj.2007 (PMC4888286; doi:10.7717/peerj.2007)
Supplement: File S3 [file peerj-04-2007-s005.zip › perl_scripts/QP/FigQpaper/Q.pdf]

1 Question set

3) **The initial velocity of a ball of mass 1 kg is 9 metre/s, and after 12 secs the final velocity is 10 metre/sec. What is the rate of change in momentum?**
4) **A 12 kg rifle fires a 10 g bullet at a velocity of 9 m/s find the recoil velocity of the rifle?**
5) **An airplane accelerates down a runway at 5 m/s*s for 7 s until is finally lifts off the ground. Determine the distance traveled before takeoff**
6) **In order to gain a velocity of 10 m/s how long should a force of 4 N be exerted on a body of mass 5 kg that is initially at rest?**

2 Question set

10) **The initial velocity of a ball of mass 13 kg is 3 metre/s, and after 1 secs the final velocity is 9 metre/sec. What is the rate of change in momentum?**
11) **A 10 kg rifle fires a 4 g bullet at a velocity of 7 m/s find the recoil velocity of the rifle?**
12) **An airplane accelerates down a runway at 3 m/s*s for 14 s until is finally lifts off the ground. Determine the distance traveled before takeoff**
13) **In order to gain a velocity of 12 m/s how long should a force of 13 N be exerted on a body of mass 4 kg that is initially at rest?**

3 Question set

17) **The initial velocity of a ball of mass 5 kg is 13 metre/s, and after 14 secs the final velocity is 15 metre/sec. What is the rate of change in momentum?**
18) **A 1 kg rifle fires a 7 g bullet at a velocity of 6 m/s find the recoil velocity of the rifle?**
19) **An airplane accelerates down a runway at 7 m/s*s for 9 s until is finally lifts off the ground. Determine the distance traveled before takeoff**
20) **In order to gain a velocity of 9 m/s how long should a force of 5 N be exerted on a body of mass 12 kg that is initially at rest?**

ANSWERS id=97814 3
==================================
2) **14 (kg*metre)/sec**
3) **0.0833333333333333 metre/(sec*sec)**
4) **7.5**
5) **122.5**
6) **12.5**
ANSWERS id=97814 2
==================================
9) **112 (kg*metre)/sec**
10) **78 metre/(sec*sec)**
11) **2.8**
12) **294**
13) **3.69230769230769**
ANSWERS id=97814 1
==================================
16) **-99 (kg*metre)/sec**
17) **0.714285714285714 metre/(sec*sec)**
18) **42**
19) **283.5**
20) **21.6**
