# Supplementary material for: Sequence/structural analysis of xylem proteome emphasizes pathogenesis-related proteins, chitinases and β-1, 3-glucanases as key players in grapevine defense against Xylella fastidiosa
Source: PeerJ. 2016 May 24;4:e2007. doi: 10.7717/peerj.2007 (PMC4888286; doi:10.7717/peerj.2007)
Supplement: File S3 [file peerj-04-2007-s005.zip › perl_scripts/QP/FigQpaper/document.pdf.orf]

BIOINFORMATICS

Vol. 00 no. 00 2010
Pages 1–10

Promiscuous metallo-β-lactamase activity in Alkaline phosphatases - a case for convergent evolution?

Sandeep Chakraborty, *Renu Minda, Lipika Salaye, Basuthkar J. Rao.

Department of Biological Sciences, Tata Institute of Fundamental Research, Homi Bhabha Road, Mumbai 400 005, India.

ABSTRACT

Motivation: Metallo-β-lactamases (MBL) present a worrisome situation as they can degrade all classes of β-lactams except monobactams and are not susceptible to therapeutic β-lactamase inhibitors. Lately there has been a marked increase in spread of the acquired or transferable families of MBLs located within a variety of integron structures. In order to trace the evolutionary origin of this function, finding enzymes with the same catalytic activity or topology using structural and sequence alignment techniques is an useful exercise. The MBL superfamily is the result of such an effort. Convergent evolution based on modular construction of active site would not be in the purview of such methods.

Results: Previous work in our group has established a modular method to detect active sites in proteins using spatial and electrostatic properties of the catalytic residues.We thus unravelled a promiscuous protease function in shrimp alkaline phosphatase. Spurred by the fact that APs, like MBLs, are metallo enzymes requring zinc atoms, we probed APs using the MBL active site motif. The results indicated that APs have MBL activity. This has been experimentally verified using microbiological assays and microiodometric data. The promisicuity of the AP active site in catalyzing the antibiotics has been demonstrated by the competitive inhibition of the AP activity of APs in the presence of antibiotics. An evolutionary linkage can be drawn between APs and MBLs based on the analogous activities in their superfamilies - phosphodiesterases, arylsulfatases and now metallo-β-lactamases. The lack of sequence homology between these superfamilies favors a convergent evolution hypothesis. Can one conjecture that this mapping between these functions in the superfamilies is complete? If so, one can predict the existence of a RNA binding enzyme with endonuclease activity corresponding to the the MBL superfamily protein tRNase Z in the AP superfamily.

Conclusion: MBLs has three subgroups based on sequence homology. Since APs possess MBL activity but do not have significant homology with any of these classes, we propose to extend the subgrouping to B4 with the APs as their first members.

Contact: sandeepchak@tifr.res.in

*To whom correspondence should be addressed: Department of Biological Sciences, Tata Institute of Fundamental Research, Colaba, Mumbai 400005, India. Phone: 91 22 22782606; fax 91 22 22804610; e-mail: sandeepchak@tifr.res.in

1 BACKGROUND

Bacteria develop resistance to β-lactam antibiotics using various mechanisms (Wilke *et al.*, 2005). Production of β-lactamase enzymes is the most common mechanism (Livermore, 1995; Bradford, 2001). Others include mutations in the active site of penicillin-binding proteins (PBP) which lower the affinity for β-lactam antibiotics, decreased expression of outer membrane proteins and efflux pumps which export substrates from the periplasm to the environment (Livermore, 1988; Amyes, 2003).

β-lactamases enzymes inactivate the antibiotics by hydrolysing the amide bond of the β-lactam ring. The Ambler classificaton has four classes - Classes A, C and D have a nucleophilic serine at the active site, while metallo-β-lactamases (MBL) or Class B β-lactamases are metallo-enzymes requiring zinc for their activity (Ambler, 1980; Bush and Jacoby, 2010). MBLs can degrade all classes of β-lactams except monobactams and are special for their constant and efficient carbapenemase activity (Bebrone, 2007). This is a most disconcerting characteristic because carbapenems are stable against the vast majority of serine-β-lactamases produced by resistant pathogens (Livermore and Woodford, 2000; Poirel *et al.*, 2007; Bebrone, 2007; Queenan and Bush, 2007). Moreover, MBLs are not susceptible to therapeutic β-lactamase inhibitors (Buynak, 2006; Drawz and Bonomo, 2010; Oelschlaeger *et al.*, 2010). MBLs have been further divided into three subgroups - B1, B2, and B3 - based on sequence homology (Rasmussen and Bush, 1997; Garau *et al.*, 2004, 2005b). Enzymes of subclass B2 selectively hydrolyze carbapenems, whereas enzymes of subclasses B1 and B3 also hydrolyze penicillins and cephalosporins (Bebrone, 2007). The striking diversity in terms of substrate spectrum, active site structure, and metal ion requirements is exemplified by the B3 GOB enzyme (Moran-Barrio *et al.*, 2007; Lisa *et al.*, 2010) in which the active form of the enzyme contains only one zinc ion. In order to find homologous enzymes, current techniques like PSI-BLAST (Altschul *et al.*, 1997) or genome database mining (Stoczko *et al.*, 2008) are limited by their ability to identify these enzymes that have conserved sequences. The MBL superfamily is the result of such a PSI-BLAST search and includes enzymes like arylsulfatases, phosphodiesterases and endonucleases (Aravind, 1999; Daiyasu *et al.*, 2001).

The phenomenon of convergent evolution, first detected in serine proteases (Kraut, 1977; Rawlings and Barrett, 1993), is no longer considered as an unique event (Doolittle, 1994; Gherardini *et al.*, 2007). Modular construction from smaller subsytems is now widely considered the underlying basis for complex and diverse organisms (Carroll, 2001; Gherardini *et al.*, 2010). We

© Oxford University Press 2010.

have established a computational method (CLASP) based on spatial and electrostatic properties for detection of active sites, as well as classifying a protein of unknown function by probing it with mulitiple motifs (**?**). Such a modular approach to query a protein has been previously adopted using only spatial congruence, which has concluded that alkaline phosphatases (AP) and aminopeptidases have evolved convergently (Russell, 1998). A recent method, ProBis, detects structurally similar sites on protein surfaces by a local structral alignment technique (Konc and Janezic, 2010), and compares its results with similar structural alignment methods (Holm *et al.*, 2008; Angaran *et al.*, 2009; Shulman-Peleg *et al.*, 2008). Although ProBis detected the relationship between alkaline phosphatases and aminopeptidases, and a weaker relationship between MBLs and adenosine deaminases, it did not report any similarity between APs and MBLs. Another innovative method (Hermann *et al.*, 2007) tries to dock high-energy intermediates of various metabolites listed in a database (Wixon and Kell, 2000) and successfully predicts the function of an unknown protein. CLASP was applied on APs, one of the widely studied promiscuous enzymes (van Loo *et al.*, 2010), which led to the discovery of a proimiscuous protease activity in shrimp alkaline phosphotase (de Backer *et al.*, 2002).

In the present work, we probed for any existing relationship between MBLs and APs by querying the MBL active site motif in the AP structure. Such an effort led us to hypothesize APs may have MBL activity. Within the MBL superfamily, true MBLs are those proteins which have biologically detectable β-lactam-hydrolyzing activity, while the others are referred to as homologs (Hall *et al.*, 2004). We have experimentally demonstrated MBL activity in APs, thus establishing it as a true MBL. To the best of our knowledege, the only relationship shown between MBLs and APs is the hydrolysis of benzylpenicillin by a complex that functionally mimics APs (Koike and Kimura, 1991; Koike *et al.*, 1994; Tamilselvi and Mugesh, 2008).

MBLs and APs can also be associated through their superfamilies. An intriguing observation is that these superfamilies have analogous enzymes, ie enzymes that that have the same activity - phosphodiesterases, arylsulfatases and, as shown in the current work, metallo-β-lactamases (Aravind, 1999; Galperin *et al.*, 1998). Since the proteins across the superfamilies do not have significant sequence homology, one is inclined to believe that these superfamilies have evolved convergently. Such congervence resulting in two structural configurations achieving the same catalytic mechanism has been previously reported (Graumann and Marahiel, 1996; Pazy *et al.*, 2010).

Furthermore, the MBL activity in these APs were not susceptible to metal chelators. Interestingly another B3 eznyme, BJP-1, was shown to be resistant to to metal chelators in contrast to other MBLs (Docquier *et al.*, 2010). Finally, since the active site in APs is responsible for both AP and MBL activities, we have shown that the AP activity is competitively inhibited in the presence of antibiotics. Since APs bear no sequence identities with the either B1, B2 or B3 classes, we propose to introduce a new subgroup B4 with the APs as its first member (Table 1).

2 RESULTS

We first describe the critical residues involved in the catalysis in the different groups of MBL. A set of these critical residues is used to define the active site motif of MBLs. The spatial and electrostatic potential difference congruence in APs to the active site in MBLs provides the rationale for proposing that these proteins might confer resistance to antibiotics. *In vitro* experiments, microbiological assays and microiodometric data, have demonstrated that this is indeed true. Finally, as an additional experimental evidence that the the highly promiscuous active site of AP is responsible for both the AP and MBL activity, the competitive inhibition of AP activity in the presence of antibiotics has been demonstrated.

2.1 Selecting the active site motif for CLASP

MBL enzymes in Classes B1 and B3 possess a binuclear active site, which requires one or two Zn^{2+} ions for full activity (Heinz and Adolph, 2004; Llarrull *et al.*, 2007; Spencer *et al.*, 2005; Jacquin *et al.*, 2009). Subclass B2 enzymes are catalytically active with one Zn^{2+} ion (Garau *et al.*, 2005a), while the binding of the second zinc ion has been shown to have inhibitory effects (Bebrone *et al.*, 2009). The Zn1 site comprises of three histidine residues (HHH site), whereas the Zn2 site is coordinated by aspartic acid, cysteine/histidine, and a histidine residue (DCH site) (Hu *et al.*, 2008). In the case of B1 enzymes, the zinc ions have a a tetrahedral coordination sphere (the histidine site - His116, His118, His196 and a water molecule or hydroxide ion) and a trigonal-pyramidal coordination sphere (the cystenine site - Asp120, Cys221, His263 and two water molecules). One water/hydroxide molecule serves as a ligand for both metal ions. In subclass B3, the histidine site is similar to that found in subclass B1, but Cys221 is replaced by a serine residue and the second zinc ion is coordinated by Asp120, His121, His263. In contrast to other B3 enzymes, GOB-18 is a mono-metallic MBL, with the catalytic Zn^{2+} ion bound to the DCH site.

In Class B2 enzymes the first zinc ion binds in the cysteine site while the second zinc ion occupies a slightly modified histidine site, where the conserved His118 and His196 residues act as metal ligands. The second zinc ion immobilizes of the catalytically important His118 and His196 residues.

In order to specify a single motif for querying MBL activity in any protein we chose the residues - {His116,His118,His196,Asp120, Cys121,His263} from a MBL (PDB id: 1ZNB) as the reference motif. While searching for matches, we allowed the His116 to be matched by either His or Gln and Cys221 to be matched with either Cys, Ser or His to accomodate the variability seen in various MBLs.

2.2 Spatial and electrostatic congruence of APs to MBLs in their active sites:

2.2.1 Metallo-β-lactamases: Table 2 shows the residues predicted by CLASP in various APs and MBLs using the MBL motif described above. For the B1 group MBLs with PDB identifiers 3FCZ and 1DD6, the predicted residues have been verified to be correct. For, the B2 MBL (PDB id: 3F90), the active site zinc is coordinated by Asp121,Cys221 and His263. The CLASP predicted active site for this protein does not match with this site. This is not surprising as this protein requires one zinc for catalysis and the other zinc is known to be inhibitory (Bebrone *et al.*, 2009). In

the FEZ-1 B3 MBL (PDB id: 1JT1), CLASP predicts all residues in concurrence with the known active site, except for one residue Ser262 in place of the His121. Similarly, Ser202 is obtained as a better match then the experimentally verified His242 in BJP-1 B3 MBL (PDB id: 3LVZ), and in the L1 B3 MBL (PDB id: 1SML) in which Ser185 is predicted in place of the His89. It is to be noted here that CLASP searches for the B1 motif - and perhaps, this configuration of a serine in place of the histidine in B3 MBLs matches better. The difference in these two classes is presumably a consequence of evolution resulting in altered specificity towards β-lactams by active site amino acid restructuring. Table 3 shows the deviations in the distances between the residues predicted by CLASP from the distances in the reference motif. As expected the deviations in the distances between the residues predicted in the proteins in the B1 are all less than 1Å. Although these deviations are also within in reasonable limits for most residue pairs in B3 proteins, some distance pairs deviate as much as 3.6Å. Deviations in these distances in B2 proteins are higher. A similar trend is seen for potential difference between pairs of residues (Table 4).

2.2.2 Alkaline Phosphatases: ECAP consists of 3 metal binding sites - M1, M2 and M3. The sites M1 and M2 bind zinc ions, while M3 binds a magnesium ion (Fig 2a). The predicted residues in ECAP coincides with M1 and M2. The deviations in the distances from the reference motif (Table 3) and potentials differences between residue pairs (Table 4) suggests that ECAP might have MBL activity. SAP consists of 3 metal binding sites, all of which bind zinc ions(Fig 2b). Unlike ECAP, the predicted residues in SAP coincide with M2 and M3. The deviations in the distances from the reference motif and potentials differences between residue pairs also deviate more than ECAP, indicating that SAP might be a weaker MBL than ECAP. The discrepancy in the sites identified in ECAP (M1,M2) and SAP (M2,M3) might also be explained by the fact that M3 in ECAP is preferably bound to a Mg, whereas in SAP this is bound to a Zn (de Backer *et al.*, 2002). Calf intestinal alkaline phosphatase could not be probed since there are no structures solved for this enzyme.

2.3 *In vitro* experiments

3 METHODS

3.0.1 Tools: The CLASP algorithm has been previously decribed (**?**). Adaptive Poisson-Boltzmann Solver (APBS) package was used to calculate the potential difference between the reactive atoms of the corresponding proteins (Baker *et al.*, 2001). The PDB2PQR package (Dolinsky *et al.*, 2004) was used to calculate the charges on the atoms using the PARSE force field (Sitkoff *et al.*, 1994). Apart from computing the charge on atoms, PDB2PQR also calculates the center of the protein which is required by APBS to apply a finite difference method to solve the Poisson-Boltzmann equation. The dielectric of the protein and water is taken as 2 and 78 respectively. We extensively integrated and used the freely available BioPerl (Stajich *et al.*, 2002) modules and Emboss (Rice *et al.*, 2000) tools. 3D structure was aligned by the TopMatch server Sippl and Wiederstein (2008). All protein structures were rendered by PyMol (http://www.pymol.org/).

3.0.2 Protein, Substrate and Reagents: SAP was purchased from USB, CIAP from Gibco BRL and ECAP from Sigma. PMSF (Phenylmethylsulfonyl fluoride) and trypsin inhibitor from chicken egg white was obtained from Roche. pNPP (p-Nitrophenyl phosphate Disodium salt Hexahydrate) was obtained from SRL. We used trypsin from Sigma.

3.0.3 β-lactamase Assay

3.0.4 Alkaline Phosphatase Assay Reaction of AP (1 unit) with p-nitrophenylphosphate (0.2 mM) as substrate, in a total volume of 200 μl was set up in 50 mM ammonium bicarbonate buffer, and incubated at room temperature for 2 min. Absorbance at 405 nm was measured against deionized water. Inhibition studies were performed as mentioned above for protease assay.

4 DISCUSSION

Zinc, an essential trace element, is found in a variety of biological applications due to its flexibility in the coordination geometry, the ability to participate in strong but readily exchangeable ligand binding and lack of redox properties which alleviates the risk of oxidative damage in living systems (Vallee and Auld, 1993; Coleman, 1998; McCall *et al.*, 2000). Another important feature is the low difference between 4, 5 or 6 coordination geometries and the fast exchange of the zinc-bound water molecule. MBLs (MBL,EC 3.5.2) and Alkaline Phosphatases (AP,EC 3.1.3.1) are two such enzyme classes where the combination of zinc and protein ligands activate a coordinated water as a nucleophile. The only known relationship shown between MBLs and APs to the best of our knowledge is that a functional complex that mimics AP can hydrolyze benzylpenicillin (Koike and Kimura, 1991; Koike *et al.*, 1994; Tamilselvi and Mugesh, 2008). In the present work, we have computationally predicted that APs might have MBL activity using CLASP (**?**). This prediction has been validated by microbiological assays and microiodometric data. The predicted catalytic residues for the MBL activity in APs overlap with the active site for its AP activity, a fact validated by the inhibition of AP activity by the addition of antibiotics.

The MBL (Aravind, 1999; Daiyasu *et al.*, 2001) and AP (Galperin *et al.*, 1998; Millan, 2006) superfamilies have been established based on sequence alignment and have proteins that often show strong structural and functional relationships. Since there is little sequence similarities across these superfamilies, we are inclined to believe that they have evolved convergently. Currently researches opine that converent evolution, first detected in serine proteases, is not that rare. Modular construction of enzymes using reiterated subsystems is the underlying feature in the evolution of such diverse and complex organisms. Techniques that use modular motifs to probe for similar structural solutions in proteins are currently used to supplement sequence alignment techniques. One such method concludes that APs and aminopeptidases have evolved convergently. A similar conclusion was drawn for the RNA-binding domain (RBD) and the cold-shock domain (CSD) protein family (Graumann and Marahiel, 1996).

one can identify several analogous functions in these superfamilies (Fig 1). Phosphodiesterases are one such enzyme category that features in both superfamilies - tRNase Z and nucleotide pyrophosphatases/phosphodiesterases (NPP) in MBL and AP superfamilies respectively (Hartmann *et al.*, 2009; Gijsbers

et al., 2001). tRNase Z, which is encoded by the ELAC gene (Vogel *et al.*, 2002), was analyzed to have homology with an arylsulfatase (Barbeyron *et al.*, 1995). Arylsulfatases are known to be a member of the AP superfamily (O'Brien and Herschlag, 1998). APs themselves are known to be weak phosphodiesterases (O'Brien and Herschlag, 2001) and arylsulfatases (Catrina *et al.*, 2007). The MBL activity detected in APs in the current work is another feature uniting thse superfamilies. While tRNase Z shows a significant topology match with MBLs (SI Fig 1a), and NPPs with APs (SI Fig 1b), they show very little similarity with each other (SI Fig 1c). We believe that these superfamilies are a consequence of convergent evolution (Doolittle, 1994; Gherardini *et al.*, 2007).

The overall topology of these enzymes are quite different too. MBLS exhibit an $\alpha\beta\beta\alpha$ fold consisting of a set of external α-helices, two internal β-sheets, and a mono or binuclear metal binding site (Fig 3a). The AP structure is divided into two domains, a core domain and a crown domain (Fig 3b). The core domain in each subunit consists of a central β-sheet running through the dimeric structure with helices packed on either site. The two β-sheets in the dimer appears like one sheet slicing through the entire molecule. The crown domains constructed of helices and one connecting loop is at the top of the molecule.

The mechanistic details of catalysis show striking similarity. The inactivation of β-lactams by MBLs involves a covalent bond formation between the carbonyl carbon of β-lactam and the attacking nucleophile followed by the C-N bond fission of the β-lactam. There are two proposed mechanisms by which the nucleophilic attack takes place. One mechanism suggests that the metal-ion acts as a Lewis acid by liganding to the peptide carbonyl oxygen, thus stabilizing the negative charge on the carbonyl carbon which facilitates nucleophilic attack. The other mechanism proposes that the water molecule directly coordinated to the Zn^{2+} ion acts as the nucleophile to attack the carbonyl carbon. Similary, the C-N bond fission has two possible mechanisms, one proposing that the departing amine nitrogen directly coordinates with the metal-ion. This is the mechanism suggested for the Zn^{2+} ion catalysed hydrolysis of penicillin (Gensmantel *et al.*, 1980). The other proposed mechanism suggest that a metal liganded water acts as a general acid catalyst by donating a proton to the amine nitrogen leaving group to aid C-N bond fission. The exact roles of the metal-ion in MBLs remain controversial despite intense mechanistic studies (Page and Badarau, 2008; Oelschlaeger, 2008; Simona *et al.*, 2009). The proposed mechanism of two-metal ion catalysis in the hydrolysis of phosphate monoesters by AP first proceeds by the formation of the enzyme-substrate complex (Coleman, 1992; Holtz and Kantrowitz, 1999; Stec *et al.*, 2000). This results from the coordination of the ester oxygen to Zn1. Phosphate now comes to occupy the active site cavity and interacts with Arg166 and the zinc ions. At alkaline pH, a nucleophilic hydroxide coordinated to Zn1 attacks the covalent intermediate forming a non-covalent complex, from which slow dissociation of phosphate occurs. This is the rate limiting step.

The physiological significance of APs exhibiting MBL activity is thought provoking. The ubiquitous presence of inducible genes carrying MBLs in certain bacteria has been attributed to various reasons. Exposure to β-lactams or β-lactam-type compounds might lead to acquistions and maintenannce of these genes and their products (Walsh *et al.*, 2005) due to selective pressures. The real physiological roles of CAU-1, BJP-1 and CAR-1 MBLs remains enigmatic since these are not produced in the presence of β-lactam antibiotics (Docquier *et al.*, 2002, 2010; Stoczko *et al.*, 2008). Virulence factors are a fascinating aspect where one can associate β-lactamases and alkaline phosphatases. Various virulence factors have been shown to be associated with outer membrane vesicles (OMVs) liberated from several pathogens (Bomberger *et al.*, 2009; Ellis and Kuehn, 2010; Amano *et al.*, 2010). OMVs contains enzymes like proelastase, hemolytic phospholipase C, hemolysins, β-lactamase, and alkaline phosphatases. One could hypothesize that such virulence factor dependent selectivity in APs led to their retaining the β-lactamase activity. APs are also known to be related to another virulence factor - biofilms (Huang *et al.*, 1998; Xu *et al.*, 1998). Recently it has been shown that β-lactamases of Class A and D inhibit bacterial biofilm formation (Gallant *et al.*, 2005). This is in agreement with the metal dependent β-lactamase that APs possess, since Class B β-lactamases did not inhibit the formation of biofilms.

Finally, in light of its MBL activity, one needs to assign an appropriate position to APs in the β-lactamase family. Classes A, C and D has a nucleophilic serine at the active site , while class B β-lactamases (MBLs) are metallo-enzymes requiring zinc ions for their activity according to the Ambler classificaton (Ambler, 1980; Bush and Jacoby, 2010). MBLs have been further divided into three subgroups - B1, B2, and B3 - based on sequence homology (Rasmussen and Bush, 1997; Garau *et al.*, 2004, 2005b). Origins of Subgroup Bl+B2 and Subgroup B3 are shown to have independent origins (Hall *et al.*, 2003, 2004). APs does not have significant homology with any of these classes (Table 1). Hence we propose to extend this classification to B4 and include APs as the first members of this subclass.

4.1 Conclusions:

5 CONCLUSION

5.0.1 Future Directions:

6 LIST OF ABBREVIATIONS

7 AUTHORS' CONTRIBUTIONS

8 ACKNOWLEDGEMENTS AND FUNDING

REFERENCES

Altschul, S. F., Madden, T. L., Schaffer, A. A., Zhang, J., Zhang, Z., Miller, W., and Lipman, D. J. (1997). Gapped BLAST and PSI-BLAST: a new generation of protein database search programs. *Nucleic Acids Res.*, **25**, 3389–3402.

Amano, A., Takeuchi, H., and Furuta, N. (2010). Outer membrane vesicles function as offensive weapons in host-parasite interactions. *Microbes Infect.*, **12**, 791–798.

Ambler, R. P. (1980). The structure of beta-lactamases. *Philos. Trans. R. Soc. Lond., B, Biol. Sci.*, **289**, 321–331.

Amyes, S. G. (2003). Resistance to beta-lactams–the permutations. *J Chemother*, **15**, 525–535.

Angaran, S., Bock, M. E., Garutti, C., and Guerra, C. (2009). MolLoc: a web tool for the local structural alignment of molecular surfaces. *Nucleic Acids Res.*, **37**, W565–570.

Aravind, L. (1999). An evolutionary classification of the metallo-beta-lactamase fold proteins. *In Silico Biol*, **1**(2), 69–91.

Baker, N. A., Sept, D., Joseph, S., Holst, M. J., and McCammon, J. A. (2001). Electrostatics of nanosystems: application to microtubules and the ribosome. *Proc. Natl. Acad. Sci. U.S.A.*, **98**, 10037–10041.

Barbeyron, T., Potin, P., Richard, C., Collin, O., and Kloareg, B. (1995). Arylsulphatase from Alteromonas carrageenovora. *Microbiology (Reading, Engl.)*, **141 (Pt 11)**, 2897–2904.

Bebrone, C. (2007). Metallo-beta-lactamases (classification, activity, genetic organization, structure, zinc coordination) and their superfamily. *Biochem. Pharmacol.*, **74**, 1686–1701.

Bebrone, C., Delbruck, H., Kupper, M. B., Schlomer, P., Willmann, C., Frere, J. M., Fischer, R., Galleni, M., and Hoffmann, K. M. (2009). The structure of the dizinc subclass B2 metallo-beta-lactamase CphA reveals that the second inhibitory zinc ion binds in the histidine site. *Antimicrob. Agents Chemother.*, **53**, 4464–4471.

Bomberger, J. M., Maceachran, D. P., Coutermarsh, B. A., Ye, S., O'Toole, G. A., and Stanton, B. A. (2009). Long-distance delivery of bacterial virulence factors by Pseudomonas aeruginosa outer membrane vesicles. *PLoS Pathog.*, **5**, e1000382.

Bradford, P. A. (2001). Extended-spectrum beta-lactamases in the 21st century: characterization, epidemiology, and detection of this important resistance threat. *Clin. Microbiol. Rev.*, **14**, 933–951.

Bush, K. and Jacoby, G. A. (2010). Updated functional classification of beta-lactamases. *Antimicrob. Agents Chemother.*, **54**, 969–976.

Buynak, J. D. (2006). Understanding the longevity of the beta-lactam antibiotics and of antibiotic/beta-lactamase inhibitor combinations. *Biochem. Pharmacol.*, **71**, 930–940.

Carroll, S. B. (2001). Chance and necessity: the evolution of morphological complexity and diversity. *Nature*, **409**, 1102–1109.

Catrina, I., O'Brien, P. J., Purcell, J., Nikolic-Hughes, I., Zalatan, J. G., Hengge, A. C., and Herschlag, D. (2007). Probing the origin of the compromised catalysis of E. coli alkaline phosphatase in its promiscuous sulfatase reaction. *J. Am. Chem. Soc.*, **129**, 5760–5765.

Coleman, J. E. (1992). Structure and mechanism of alkaline phosphatase. *Annu Rev Biophys Biomol Struct*, **21**, 441–483.

Coleman, J. E. (1998). Zinc enzymes. *Curr Opin Chem Biol*, **2**, 222–234.

Daiyasu, H., Osaka, K., Ishino, Y., and Toh, H. (2001). Expansion of the zinc metallo-hydrolase family of the beta-lactamase fold. *FEBS Lett.*, **503**, 1–6.

de Backer, M., McSweeney, S., Rasmussen, H. B., Riise, B. W., Lindley, P., and Hough, E. (2002). The 1.9 A crystal structure of heat-labile shrimp alkaline phosphatase. *J. Mol. Biol.*, **318**, 1265–1274.

Docquier, J. D., Pantanella, F., Giuliani, F., Thaller, M. C., Amicosante, G., Galleni, M., Frere, J. M., Bush, K., and Rossolini, G. M. (2002). CAU-1, a subclass B3 metallo-beta-lactamase of low substrate affinity encoded by an ortholog present in the Caulobacter crescentus chromosome. *Antimicrob. Agents Chemother.*, **46**, 1823–1830.

Docquier, J. D., Benvenuti, M., Calderone, V., Stoczko, M., Menciassi, N., Rossolini, G. M., and Mangani, S. (2010). High-resolution crystal structure of the subclass B3 metallo-beta-lactamase BJP-1: rational basis for substrate specificity and interaction with sulfonamides. *Antimicrob. Agents Chemother.*, **54**, 4343–4351.

Dolinsky, T. J., Nielsen, J. E., McCammon, J. A., and Baker, N. A. (2004). PDB2PQR: an automated pipeline for the setup of Poisson-Boltzmann electrostatics calculations. *Nucleic Acids Res.*, **32**, W665–667.

Doolittle, R. F. (1994). Convergent evolution: the need to be explicit. *Trends Biochem. Sci.*, **19**, 15–18.

Drawz, S. M. and Bonomo, R. A. (2010). Three decades of beta-lactamase inhibitors. *Clin. Microbiol. Rev.*, **23**, 160–201.

Ellis, T. N. and Kuehn, M. J. (2010). Virulence and immunomodulatory roles of bacterial outer membrane vesicles. *Microbiol. Mol. Biol. Rev.*, **74**, 81–94.

Gallant, C. V., Daniels, C., Leung, J. M., Ghosh, A. S., Young, K. D., Kotra, L. P., and Burrows, L. L. (2005). Common beta-lactamases inhibit bacterial biofilm formation. *Mol. Microbiol.*, **58**, 1012–1024.

Galperin, M. Y., Bairoch, A., and Koonin, E. V. (1998). A superfamily of metalloenzymes unifies phosphopentomutase and cofactor-independent phosphoglycerate mutase with alkaline phosphatases and sulfatases. *Protein Sci.*, **7**, 1829–1835.

Garau, G., Garcia-Saez, I., Bebrone, C., Anne, C., Mercuri, P., Galleni, M., Frere, J. M., and Dideberg, O. (2004). Update of the standard numbering scheme for class B beta-lactamases. *Antimicrob. Agents Chemother.*, **48**, 2347–2349.

Garau, G., Bebrone, C., Anne, C., Galleni, M., Frere, J. M., and Dideberg, O. (2005a). A metallo-beta-lactamase enzyme in action: crystal structures of the monozinc carbapenemase CphA and its complex with biapenem. *J. Mol. Biol.*, **345**, 785–795.

Garau, G., Di Guilmi, A. M., and Hall, B. G. (2005b). Structure-based phylogeny of the metallo-beta-lactamases. *Antimicrob. Agents Chemother.*, **49**, 2778–2784.

Gensmantel, N. P., Proctor, P., and Page, M. I. (1980). Metal-ion catalysed hydrolysis of some [small beta]-lactam antibiotics. *J. Chem. Soc., Perkin Trans. 2*, pages 1725–1732.

Gherardini, P. F., Wass, M. N., Helmer-Citterich, M., and Sternberg, M. J. (2007). Convergent evolution of enzyme active sites is not a rare phenomenon. *J. Mol. Biol.*, **372**, 817–845.

Gherardini, P. F., Ausiello, G., Russell, R. B., and Helmer-Citterich, M. (2010). Modular architecture of nucleotide-binding pockets. *Nucleic Acids Res.*, **38**, 3809–3816.

Gijsbers, R., Ceulemans, H., Stalmans, W., and Bollen, M. (2001). Structural and catalytic similarities between nucleotide pyrophosphatases/phosphodiesterases and alkaline phosphatases. *J. Biol. Chem.*, **276**, 1361–1368.

Graumann, P. and Marahiel, M. A. (1996). A case of convergent evolution of nucleic acid binding modules. *Bioessays*, **18**, 309–315.

Hall, B. G., Salipante, S. J., and Barlow, M. (2003). The metallo-beta-lactamases fall into two distinct phylogenetic groups. *J. Mol. Evol.*, **57**, 249–254.

Hall, B. G., Salipante, S. J., and Barlow, M. (2004). Independent origins of subgroup Bl + B2 and subgroup B3 metallo-beta-lactamases. *J. Mol. Evol.*, **59**, 133–141.

Hartmann, R. K., Gossringer, M., Spath, B., Fischer, S., and Marchfelder, A. (2009). The making of tRNAs and more - RNase P and tRNase Z. *Prog Mol Biol Transl Sci*, **85**, 319–368.

Heinz, U. and Adolph, H. W. (2004). Metallo-beta-lactamases: two binding sites for one catalytic metal ion? *Cell. Mol. Life Sci.*, **61**, 2827–2839.

Hermann, J. C., Marti-Arbona, R., Fedorov, A. A., Fedorov, E., Almo, S. C., Shoichet, B. K., and Raushel, F. M. (2007). Structure-based activity prediction for an enzyme of unknown function. *Nature*, **448**, 775–779.

Holm, L., Kaariainen, S., Rosenstrom, P., and Schenkel, A. (2008). Searching protein structure databases with DaliLite v.3. *Bioinformatics*, **24**, 2780–2781.

Holtz, K. M. and Kantrowitz, E. R. (1999). The mechanism of the alkaline phosphatase reaction: insights from NMR, crystallography and site-specific mutagenesis. *FEBS Lett.*, **462**, 7–11.

Hu, Z., Periyannan, G., Bennett, B., and Crowder, M. W. (2008). Role of the Zn1 and Zn2 sites in metallo-beta-lactamase L1. *J. Am. Chem. Soc.*, **130**, 14207–14216.

Huang, C. T., Xu, K. D., McFeters, G. A., and Stewart, P. S. (1998). Spatial patterns of alkaline phosphatase expression within bacterial colonies and biofilms in response to phosphate starvation. *Appl. Environ. Microbiol.*, **64**, 1526–1531.

Jacquin, O., Balbeur, D., Damblon, C., Marchot, P., De Pauw, E., Roberts, G. C., Frere, J. M., and Matagne, A. (2009). Positively cooperative binding of zinc ions to Bacillus cereus 569/H/9 beta-lactamase II suggests that the binuclear enzyme is the only relevant form for catalysis. *J. Mol. Biol.*, **392**, 1278–1291.

Koike, T. and Kimura, E. (1991). Roles of zinc(ii) ion in phosphatases. a model study with zinc(ii)-macrocyclic polyamine complexes. *Journal of the American Chemical Society*, **113**(23), 8935–8941.

Koike, T., Takamura, M., and Kimura, E. (1994). Role of zinc(ii) in .beta.-lactamase ii: A model study with a zinc(ii)-macrocyclic tetraamine (1,4,7,10-tetraazacyclododecane, cyclen) complex. *Journal of the American Chemical Society*, **116**(19), 8443–8449.

Konc, J. and Janezic, D. (2010). ProBiS algorithm for detection of structurally similar protein binding sites by local structural alignment. *Bioinformatics*, **26**, 1160–1168.

Kraut, J. (1977). Serine proteases: structure and mechanism of catalysis. *Annu. Rev. Biochem.*, **46**, 331–358.

Lisa, M. N., Hemmingsen, L., and Vila, A. J. (2010). Catalytic role of the metal ion in the metallo-beta-lactamase GOB. *J. Biol. Chem.*, **285**, 4570–4577.

Livermore, D. M. (1988). Permeation of beta-lactam antibiotics into Escherichia coli, Pseudomonas aeruginosa, and other gram-negative bacteria. *Rev. Infect. Dis.*, **10**, 691–698.

Livermore, D. M. (1995). beta-Lactamases in laboratory and clinical resistance. *Clin. Microbiol. Rev.*, **8**, 557–584.

Livermore, D. M. and Woodford, N. (2000). Carbapenemases: a problem in waiting? *Curr. Opin. Microbiol.*, **3**, 489–495.

Llarrull, L. I., Tioni, M. F., Kowalski, J., Bennett, B., and Vila, A. J. (2007). Evidence for a dinuclear active site in the metallo-beta-lactamase BcII with substoichiometric Co(II). A new model for metal uptake. *J. Biol. Chem.*, **282**, 30586–30595.

McCall, K. A., Huang, C., and Fierke, C. A. (2000). Function and mechanism of zinc metalloenzymes. *J. Nutr.*, **130**, 1437S–46S.

Millan, J. L. (2006). Alkaline Phosphatases : Structure, substrate specificity and functional relatedness to other members of a large superfamily of enzymes. *Purinergic Signal.*, **2**, 335–341.

Moran-Barrio, J., Gonzalez, J. M., Lisa, M. N., Costello, A. L., Peraro, M. D., Carloni, P., Bennett, B., Tierney, D. L., Limansky, A. S., Viale, A. M., and Vila, A. J. (2007). The metallo-beta-lactamase GOB is a mono-Zn(II) enzyme with a novel active site. *J. Biol. Chem.*, **282**, 18286–18293.

O'Brien, P. J. and Herschlag, D. (1998). Sulfatase Activity of E. coli Alkaline Phosphatase Demonstrates a Functional Link to Arylsulfatases, an Evolutionarily Related Enzyme Family. *J. Am. Chem. Soc.*, **120**, 12369–12370.

O'Brien, P. J. and Herschlag, D. (2001). Functional interrelationships in the alkaline phosphatase superfamily: phosphodiesterase activity of Escherichia coli alkaline

phosphatase. *Biochemistry*, **40**, 5691–5699.

Oelschlaeger, P. (2008). Outsmarting metallo-beta-lactamases by mimicking their natural evolution. *J. Inorg. Biochem.*, **102**, 2043–2051.

Oelschlaeger, P., Ai, N., Duprez, K. T., Welsh, W. J., and Toney, J. H. (2010). Evolving carbapenemases: can medicinal chemists advance one step ahead of the coming storm? *J. Med. Chem.*, **53**, 3013–3027.

Page, M. I. and Badarau, A. (2008). The mechanisms of catalysis by metallo beta-lactamases. *Bioinorg Chem Appl*, page 576297.

Pazy, Y., Motaleb, M. A., Guarnieri, M. T., Charon, N. W., Zhao, R., and Silversmith, R. E. (2010). Identical phosphatase mechanisms achieved through distinct modes of binding phosphoprotein substrate. *Proc. Natl. Acad. Sci. U.S.A.*, **107**, 1924–1929.

Poirel, L., Pitout, J. D., and Nordmann, P. (2007). Carbapenemases: molecular diversity and clinical consequences. *Future Microbiol*, **2**, 501–512.

Queenan, A. M. and Bush, K. (2007). Carbapenemases: the versatile beta-lactamases. *Clin. Microbiol. Rev.*, **20**, 440–458.

Rasmussen, B. A. and Bush, K. (1997). Carbapenem-hydrolyzing beta-lactamases. *Antimicrob. Agents Chemother.*, **41**, 223–232.

Rawlings, N. D. and Barrett, A. J. (1993). Evolutionary families of peptidases. *Biochem. J.*, **290 (Pt 1)**, 205–218.

Rice, P., Longden, I., and Bleasby, A. (2000). EMBOSS: the European Molecular Biology Open Software Suite. *Trends Genet.*, **16**, 276–277.

Russell, R. B. (1998). Detection of protein three-dimensional side-chain patterns: new examples of convergent evolution. *J. Mol. Biol.*, **279**, 1211–1227.

Shulman-Peleg, A., Shatsky, M., Nussinov, R., and Wolfson, H. J. (2008). MultiBind and MAPPIS: webservers for multiple alignment of protein 3D-binding sites and their interactions. *Nucleic Acids Res.*, **36**, W260–264.

Simona, F., Magistrato, A., Dal Peraro, M., Cavalli, A., Vila, A. J., and Carloni, P. (2009). Common mechanistic features among metallo-beta-lactamases: a computational study of Aeromonas hydrophila CphA enzyme. *J. Biol. Chem.*, **284**, 28164–28171.

Sippl, M. J. and Wiederstein, M. (2008). A note on difficult structure alignment problems. *Bioinformatics*, **24**, 426–427.

Sitkoff, D., Sharp, K. A., and Honig, B. (1994). Accurate calculation of hydration free energies using macroscopic solvent models. *The Journal of Physical Chemistry*, **98**(7), 1978–1988.

Spencer, J., Read, J., Sessions, R. B., Howell, S., Blackburn, G. M., and Gamblin, S. J. (2005). Antibiotic recognition by binuclear metallo-beta-lactamases revealed by X-ray crystallography. *J. Am. Chem. Soc.*, **127**, 14439–14444.

Stajich, J. E., Block, D., Boulez, K., Brenner, S. E., Chervitz, S. A., Dagdigian, C., Fuellen, G., Gilbert, J. G., Korf, I., Lapp, H., Lehväslaiho, H., Matsalla, C., Mungall, C. J., Osborne, B. I., Pocock, M. R., Schattner, P., Senger, M., Stein, L. D., Stupka, E., Wilkinson, M. D., and Birney, E. (2002). The bioperl toolkit: Perl modules for the life sciences. *Genome research*, **12**(10), 1611–1618.

Stec, B., Holtz, K. M., and Kantrowitz, E. R. (2000). A revised mechanism for the alkaline phosphatase reaction involving three metal ions. *J. Mol. Biol.*, **299**, 1303–1311.

Stoczko, M., Frere, J. M., Rossolini, G. M., and Docquier, J. D. (2008). Functional diversity among metallo-beta-lactamases: characterization of the CAR-1 enzyme of Erwinia carotovora. *Antimicrob. Agents Chemother.*, **52**, 2473–2479.

Tamilselvi, A. and Mugesh, G. (2008). Zinc and antibiotic resistance: metallo-beta-lactamases and their synthetic analogues. *J. Biol. Inorg. Chem.*, **13**, 1039–1053.

Vallee, B. L. and Auld, D. S. (1993). Zinc: biological functions and coordination motifs. *Accounts of Chemical Research*, **26**(10), 543–551.

van Loo, B., Jonas, S., Babtie, A. C., Benjdia, A., Berteau, O., Hyvonen, M., and Hollfelder, F. (2010). An efficient, multiply promiscuous hydrolase in the alkaline phosphatase superfamily. *Proc. Natl. Acad. Sci. U.S.A.*, **107**, 2740–2745.

Vogel, A., Schilling, O., Niecke, M., Bettmer, J., and Meyer-Klaucke, W. (2002). ElaC encodes a novel binuclear zinc phosphodiesterase. *J. Biol. Chem.*, **277**, 29078–29085.

Walsh, T. R., Toleman, M. A., Poirel, L., and Nordmann, P. (2005). Metallo-beta-lactamases: the quiet before the storm? *Clin. Microbiol. Rev.*, **18**, 306–325.

Wilke, M. S., Lovering, A. L., and Strynadka, N. C. (2005). Beta-lactam antibiotic resistance: a current structural perspective. *Curr. Opin. Microbiol.*, **8**, 525–533.

Wixon, J. and Kell, D. (2000). The Kyoto encyclopedia of genes and genomes–KEGG. *Yeast*, **17**, 48–55.

Xu, K. D., Stewart, P. S., Xia, F., Huang, C. T., and McFeters, G. A. (1998). Spatial physiological heterogeneity in Pseudomonas aeruginosa biofilm is determined by oxygen availability. *Appl. Environ. Microbiol.*, **64**, 4035–4039.

Table 1. Sequence identities/similarities between APs and MBLs. CIAP and GOB1 are not PDB identifiers, since there are no solved structures for these enzymes.

PDB	1ZNB	3FCZ	1DDK	3F9O	1JT1	1SML	3LVZ	GOB1	1ALK	1K7H	CIAP
1ZNB(B1)	-/-	31/51	35/56	30/46	20/34	18/35	22/32	20/36	13/22	13/23	14/21
3FCZ(B1)	NA	-/-	31/47	28/45	17/31	19/33	22/35	18/34	13/22	13/21	12/18
1DDK(B1)	NA	NA	-/-	24/44	21/37	21/32	21/33	21/34	11/20	12/22	11/16
3F9O(B2)	NA	NA	NA	-/-	19/35	22/37	23/38	21/36	11/19	10/18	10/16
1JT1(B3)	NA	NA	NA	NA	-/-	32/48	35/55	37/54	14/22	13/22	12/23
1SML(B3)	NA	NA	NA	NA	NA	-/-	33/49	24/43	15/22	16/25	15/22
3LVZ(B3)	NA	NA	NA	NA	NA	NA	-/-	31/45	20/31	17/26	16/25
GOB1(B3)	NA	NA	NA	NA	NA	NA	NA	-/-	16/22	11/20	10/18
1ALK(ECAP)	NA	NA	NA	NA	NA	NA	NA	NA	-/-	28/39	27/40
1K7H(SAP)	NA	NA	NA	NA	NA	NA	NA	NA	NA	-/-	37/51
CIAP	NA	NA	NA	NA	NA	NA	NA	NA	NA	NA	-/-

Table 2. Active sites predicted in various proteins by CLASP using the MBL motif {His116,His118,His196,Asp120,Cys121,His263}.His116 to be matched by either His or Gln and Cys221 to be matched with either Cys, Ser or His to accomodate the variability seen in various MBLs.

	His116	His118	His196	Asp120	Cys121	His263
	a	b	c	d	e	f
3FCZ(B1)	HIS/116	HIS/118	HIS/196	ASP/120	CYS/221	HIS/263
1DD6(B1)	HIS/77	HIS/79	HIS/139	ASP/81	CYS/158	HIS/197
3F9O(B2)	GLN/68	HIS/263	HIS/196	ASP/120	CYS/221	HIS/118
1JT1(B3)	HIS/116	HIS/118	HIS/196	ASP/120	SER/262	HIS/263
1SML(B3)	HIS/84	HIS/86	HIS/160	ASP/88	SER/185	HIS/225
3LVZ(B3)	HIS/101	HIS/103	HIS/177	ASP/105	SER/202	HIS/242
1ALK(ECAP)	HIS/331	HIS/372	HIS/412	ASP/327	HIS/370	HIS/86
1K7H(SAP)	HIS/359	HIS/357	HIS/432	ASP/37	SER/86	HIS/149

Table 3. Distances between pairs of residues obtained by searching for the motif {His116,His118,His196,Asp120,Cys121,His263} in a MBL (PDB id: 1ZNB). The distances are specified in the reference protein (PDB id: 1ZNB) in Å. For the remaining, we show the deviation from the reference value.

	ab	ac	ad	ae	af	bc	bd	be	bf	cd	ce	cf	de	df	ef
1ZNB(B1)	5.372	6.352	6.188	4.569	9.589	5.283	4.569	6.223	9.118	7.566	5.354	8.526	4.902	6.010	5.282
3FCZ(B1)	0.4	0.2	0.1	0.1	-0.4	-0.1	-0.3	0.3	-1.0	0.2	0.4	-0.3	0.5	-0.4	-0.4
1DDK(B1)	0.6	-0.3	0.6	0.1	0.6	-0.2	-0.4	0.4	-0.4	-0.5	-0.2	-0.6	0.5	0.1	0.3
3F90(B2)	-2.0	-9.5	-6.7	-7.7	-7.4	-3.5	-2.0	0.3	-0.6	0.0	-0.2	3.2	1.1	1.0	-0.6
1JT1(B3)	0.2	0.6	-0.7	0.1	1.1	0.3	-1.5	-2.2	-0.3	-0.8	-2.4	-1.1	-1.7	1.7	-0.4
1SML(B3)	0.2	0.6	-0.3	0.5	0.9	0.0	0.1	-0.8	-0.1	-0.4	0.5	-0.5	-2.4	-0.4	-0.9
3LVZ(B3)	0.3	0.5	-0.3	-0.4	1.0	0.3	-0.0	-0.4	-0.2	-0.5	2.0	-1.0	-3.6	-0.4	-2.7
1ALK(ECAP)	0.4	-0.3	0.8	-3.5	-4.2	-1.3	-0.4	0.3	-2.1	2.5	1.8	0.8	0.3	-4.6	-0.9
1K7H(SAP)	0.8	0.4	0.1	-3.2	-2.1	1.9	-0.1	1.0	-1.7	1.5	0.7	-1.6	1.5	-0.9	-0.7

Table 4. APBS calculated potential difference between pairs of residues obtained by searching for the motif -{His116,His118,His196,Asp120,Cys121,His263}

	ab	ac	ad	ae	af	bc	bd	be	bf	cd	ce	cf	de	df	ef
1ZNB(B1)	45.2	-137.7	24.9	-178.3	-101.1	-182.9	-20.3	-223.5	-146.3	162.7	-40.6	36.6	-203.2	-126.0	77.2
3FCZ(B1)	120.7	-129.7	18.7	-187.7	-114.1	-250.4	-102.0	-308.3	-234.8	148.4	-58.0	15.6	-206.4	-132.8	73.6
1DD6(B1)	182.1	-81.8	143.3	-69.6	-38.5	-263.9	-38.8	-251.7	-220.6	225.1	12.2	43.3	-212.9	-181.8	31.1
3F9O(B2)	-133.9	-207.2	-29.8	-264.5	-166.4	-73.3	104.1	-130.6	-32.5	177.4	-57.3	40.8	-234.7	-136.6	98.1
1JT1(B3)	139.2	-55.9	327.5	2.5	42.2	-195.2	188.3	-136.7	-97.0	383.5	58.5	98.1	-325.0	-285.4	39.7
1SML(B3)	138.8	-110.9	106.7	-5.1	36.7	-249.7	-32.1	-143.9	-102.1	217.6	105.8	147.6	-111.8	-70.0	41.9
3LVZ(B3)	22.3	-163.3	12.6	-145.8	-136.1	-185.6	-9.6	-168.0	-158.4	176.0	17.6	27.2	-158.4	-148.8	9.6
1ALK(ECAP)	110.7	-125.9	63.0	-176.3	-21.3	-236.6	-47.7	-287.0	-132.0	188.9	-50.5	104.6	-239.4	-84.3	155.0
1K7H(SAP)	-81.3	-128.1	201.0	-14.5	7.2	-46.8	282.3	66.7	88.5	329.1	113.6	135.3	-215.5	-193.8	21.7

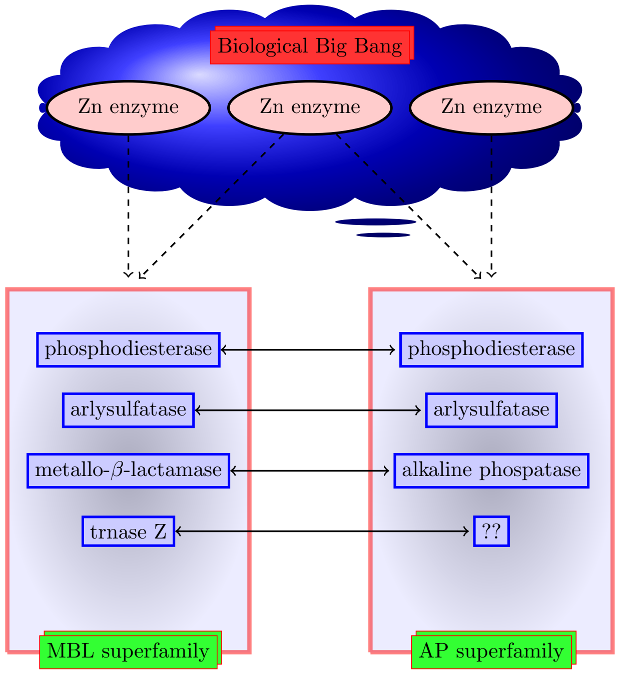

Fig. 1: The existence of analogous enzymes, ie enzymes performing the same catalytic activity, in the superfamilies of APs and MBLs suggests an evolutionary linkage - either a divergent or a convergent evolution. The lack of sequence homology across the superfamily proteins seems to favor the convergent hypothesis. Is it possible that a protein from the AP superfamily has RNA binding and endonuclease activity?

9 SUPPLEMENTARY INFORMATION: REFERENCES FROM MAIN PAPER:

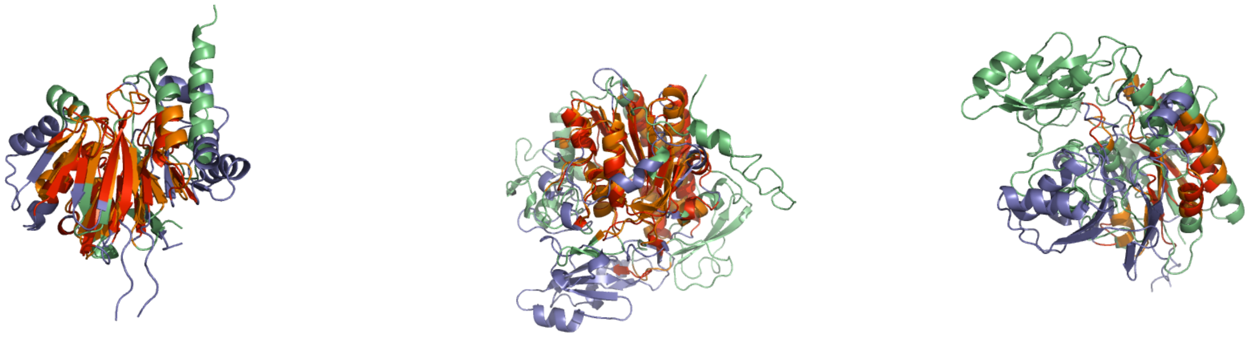

(a) (b) (c)

Supplementary Fig. 1: 3D structural alignment done using Topmatch. The query sequence is colored blue, the target sequence is colored green and pairs of structurally equivalent residues are colored red (query) or orange (target). **(a)** Query is trnase Z (PDB id: 1Y44) , Target is metallo-β-lactamase (PDB id: 1ZNB). RMS - 2.9 **(b)** Query is nucleotide pyrophosphatases/phosphodiesterases (PDB id: 2GSN) , Target is Alkaline phosphatase (PDB id: 1ALK). RMS - 3.1 **(c)** Query is trnase Z (PDB id: 1Y44) , Target nucleotide pyrophosphatases/phosphodiesterases (PDB id: 2GSN). RMS - 3.9

Table 1. Identity and Similarity among all APs

PDB id	1ED9 E. coli	1K7H Shrimp	2IUC Antarctic Bacterium	1EW2 human placenta	2X98 Halobacterium salinarum	3E2D Vibrio strain G15-2
1ED9	100/100	26.5/38.1	26.4/40.6	26.5/40.1	27.4/38.7	22.0/32.0
1K7H	-	100/100	23.5/35.6	37.2/51.4	25.8/37.5	25.0/37.4
2IUC	-	-	100/100	21.4/34.8	23.4/37.4	23.8/35.1
1EW2	-	-	-	100/100	25.9/36.4	23.7/35.2
2X98	-	-	-	-	100/100	25.0/36.7
3E2D	-	-	-	-	-	100/100

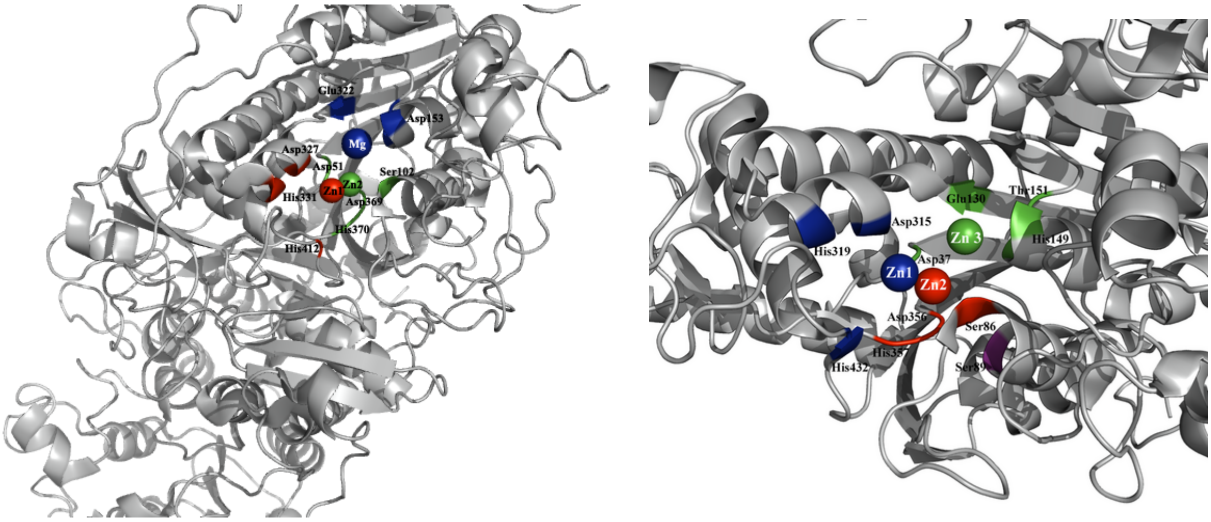

(a) (b)

Supplementary Fig. 2: **(a)** The active site for ECAP (PDB id: 1ALK) consists of 3 metal binding sites - M1 (Asp327,His331,His412) in red binds a zinc, M2 (Asp51,Ser102,Asp369,His370) in green binds another zinc, while M3 (Asp153,Thr155,Glu322) in blue binds a magnesium ion. CLASP predicted active site residues for MBL activity (HIS/331 , HIS/372 , HIS/412 , ASP/327 , HIS/370 , HIS/86) coincides with the M1 and M2 site. **(b)** The active site for SAP (PDB id: 1K7H) consists of 3 metal binding sites - M1 (Asp315,His319,His432) in blue binds a zinc, M2 (Asp37,Ser86,Asp356,His357) in red binds another zinc, while M3 (Asp37,His149,Thr151,Glu130) in green binds either a zinc or a magnesium ion. CLASP predicted active site residues for MBL activity (HIS/359 , HIS/357 , HIS/432 , ASP/37 , SER/86 , HIS/149) coincides with the M2 and M2 site. Note that this differs from the ECAP site, where M3 binds a magnesium ion, which is not conducive for MBL catalysis.

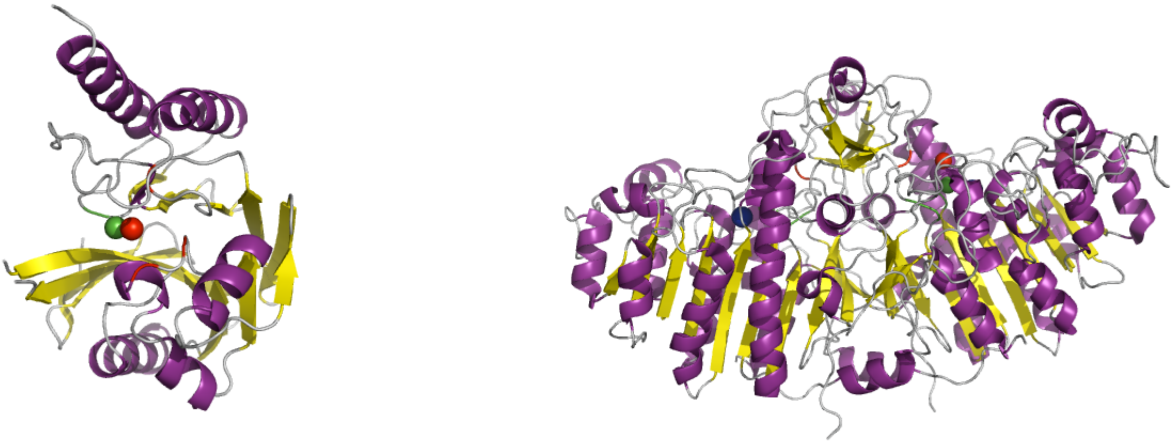

(a) (b)

Supplementary Fig. 3: **(a)** **(b)** Topology of ECAP: ECAP is dimer with a central ten-stranded β-sheet in the middle flanked by 15 helices of various lengths
