# Supplementary material for: Sequence/structural analysis of xylem proteome emphasizes pathogenesis-related proteins, chitinases and β-1, 3-glucanases as key players in grapevine defense against Xylella fastidiosa
Source: PeerJ. 2016 May 24;4:e2007. doi: 10.7717/peerj.2007 (PMC4888286; doi:10.7717/peerj.2007)
Supplement: File S3 [file peerj-04-2007-s005.zip › perl_scripts/QP/FigQpaper/document.pdf]

Template

Sandeep Chakraborty[1],
1. Department of Biological Sciences, Tata Institute of Fundamental Research, Homi Bhabha Road, Mumbai 400 005, India.

Abstract

Introduction

Class 9

1 Question set

1) **Find the sum of: - 17 + 3 + 4 - 11 - 4**
2) **210 / 14 = ?**
3) **0.4 X 1.7 = ?**
4) **10/4 + 12/5**
5) **Arrange in ascending order 1/11 , 9/6 , 8/5**
6) **2 girls can build a house in 11 days. How many days will 16 girls build it?**
7) **4 books costs Rs 7 . What is the cost of 44 books?**
8) **Find the square root of 11 - sqrt(72)**
9) **Multiply (+4a+2b) (+2a-2b)**
10) **Factorise -4aa -12ab +16bb**
11) **The volume of 9 Kg of material M1 is 2 litres, and the volume of 20 KG of material M2 is 15 litres. Which material is heavier?**
12) **The molecular mass of a compound C is 5. How many atoms are there in 18 grams of the compound?**
13) **The atomic number of an element E is 20, and its mass number is 23. How many neutrons does the element have?**
14) **Universal set = (i, o, g, v, y, j, b, s, m, c) set A = (b, o, m, s, j), set B = (g, v, c, o, j, m) . Find A intersection B by drawing venn diagrams**
15) **Factorize 125*g*g*g + 8*b*b*b**
16) **If you have scored 56 marks in History out of 120 marks, what is the percentage of marks scored by you**
17) **The angles of a triangle are in the ratio of 16, 17 and 3. Find their absolute values**
18) **According to Newton's second law, force is proportional to**
19) **The initial velocity of a ball of mass 15 kg is 2 metre/s and the final velocity is 7 metre/sec. What is the change in velocity**
20) **The initial velocity of a ball of mass 6 kg is 5 metre/s and the final velocity is 8 metre/sec. What is the change in momentum?**
21) **The initial velocity of a ball of mass 4 kg is 7 metre/s, and after 3 secs the final velocity is 13 metre/sec. What is the rate of change in momentum?**
22) **A 13 kg rifle fires a 9 g bullet at a velocity of 11 m/s find the recoil velocity of the rifle?**
23) **A 10 g bullet at a travelling at a velocity of 12 m/s hits a stationary plank of wood of mass 1 g and gets embedded in the plank. What is the velocity with which the combined mass of the wood and the bullet moves ?**
24) **An airplane accelerates down a runway at 14 m/s*s for 4 s until it finally lifts off the ground. Determine the distance traveled before takeoff**
25) **In order to gain a velocity of 9 m/s how long should a force of 2 N be exerted on a body of mass 15 kg that is initially at rest?**
26) **Radius of a circle is 42 cm. Find its area**
27) **What is the distance between the points (-2,3) and (5, 1)?**
28) **Find the coordinate of the point that divides the points (-1,-17) and (18, 8) internally in the ratio of 19:12?**
29) **What is the area of a right angled triangle with base = 5 cm and height = 7 cm ?**
30) **What is the area of a isosceles triangle with base = 6 cm and height = 1 cm ?**
31) **What is the radius of a circle with a chord = 16 cm and distance of the chord from centre = 9? cm**
32) **What number added to -10 gives 48?**

33) **What number multiplied by -10 gives 46?**
34) **What number divided by -49 gives -12?**
35) **Rishi and Pooja have some money. If you multiply Rishi 's money by 8 and Pooja 's money by 7 and add them up, you get Rs 163. If you multiply Rishi 's money by 14 and Pooja 's money by 3 and add them up, you get Rs 165. How much money does each person have?**
36) **A father is 4 times as old as his son, after 13 years, the father will be 2.7 as old as his son at that time, therefore, the fathers current age is:**
37) **Two numbers are in the ratio 6. If you add 14 to both, their ratio becomes 3.9. Find the numbers :**
38) **One piece of pipe 47 meters long is to be cut into two pieces, with the lengths of the pieces being in a 13 : 19 ratio. What are the lengths of the pieces?**
39) **Find the unknown value in the proportion: 13 : x = 19 : 5.**
40) **x is directly proportional to y. When x is 10, y is -7. Find x when y is 12.**

2 Question set

41) **Find the sum of: - 10 + 17 - 18 - 6 - 1**
42) **80 / 8 = ?**
43) **0.7 X 0.1 = ?**
44) **13/6 + 9/12**
45) **Arrange in ascending order 5/14 , 15/9 , 11/4**
46) **15 women can build a garden in 54 days. How many women will make it in 9 days ?**
47) **11 oranges costs Rs 8 . How many oranges can you buy for Rs 56?**
48) **Find the square root of 13 + sqrt(144)**
49) **Multiply (-3a+3b) (+2a+1b)**
50) **Factorise -5aa -16ab +16bb**
51) **The volume of 1 Kg of material M1 is 16 litres, and the volume of 2 KG of material M2 is 4 litres. Which material is heavier?**
52) **The molecular mass of a compound C is 24. How many atoms are there in 20 grams of the compound?**
53) **The atomic number of an element E is 20, and its mass number is 28. How many neutrons does the element have?**
54) **Universal set = (f, x, o, h, c, l, u, m, w, v) set A = (w, l, u, x, h), set B = (f, u, l, m, h, o) . Find A intersection B by drawing venn diagrams**
55) **Factorize 1*t*t*t + 8*v*v*v**
56) **If you have scored 3 marks in Geography out of 150 marks, what is the percentage of marks scored by you**
57) **The angles of a triangle are in the ratio of 6, 9 and 21. Find their absolute values**
58) **According to Newton's second law, force is proportional to**
59) **The initial velocity of a ball of mass 10 kg is 14 metre/s and the final velocity is 4 metre/sec. What is the change in velocity**
60) **The initial velocity of a ball of mass 1 kg is 8 metre/s and the final velocity is 9 metre/sec. What is the change in momentum?**
61) **The initial velocity of a ball of mass 5 kg is 2 metre/s, and after 1 secs the final velocity is 4 metre/sec. What is the rate of change in momentum?**
62) **A 10 kg rifle fires a 11 g bullet at a velocity of 15 m/s find the recoil velocity of the rifle?**
63) **A 13 g bullet at a travelling at a velocity of 15 m/s hits a stationary plank of wood of mass 12 g and gets embedded in the plank. What is the velocity with which the combined mass of the wood and the bullet moves ?**

64) **An airplane accelerates down a runway at 10 m/s*s for 3 s until it finally lifts off the ground. Determine the distance traveled before takeoff**
65) **In order to gain a velocity of 7 m/s how long should a force of 2 N be exerted on a body of mass 15 kg that is initially at rest?**
66) **Radius of a circle is 35 cm. Find its area**
67) **What is the distance between the points (5,-2) and (-4, 6)?**
68) **Find the coordinate of the point that divides the points (-8,-9) and (12, -6) internally in the ratio of 13:19?**
69) **What is the area of a right angled triangle with base = 6 cm and height = 13 cm ?**
70) **What is the area of a isosceles triangle with base = 13 cm and height = 15 cm ?**
71) **What is the radius of a circle with a chord = 8 cm and distance of the chord from centre = 20? cm**
72) **What number added to 39 gives -23?**
73) **What number multiplied by -3 gives 31?**
74) **What number divided by -50 gives 38?**
75) **Amba and Ravi have some money. If you multiply Amba 's money by 6 and Ravi 's money by 8 and add them up, you get Rs 130. If you multiply Amba 's money by 15 and Ravi 's money by 11 and add them up, you get Rs 253. How much money does each person have?**
76) **A father is 5 times as old as his son, after 11 years, the father will be 3.5 as old as his son at that time, therefore, the fathers current age is:**
77) **Two numbers are in the ratio 6. If you add 14 to both, their ratio becomes 3.5. Find the numbers :**
78) **One piece of pipe 42 meters long is to be cut into two pieces, with the lengths of the pieces being in a 19 : 4 ratio. What are the lengths of the pieces?**
79) **Find the unknown value in the proportion: 10 : x = -16 : -19.**
80) **x is directly proportional to y. When x is 4, y is -19. Find x when y is 18.**

3 Question set

81) **Find the sum of: - 13 + 5 - 20 + 12 + 3**
82) **126 / 9 = ?**
83) **1.8 X 0.2 = ?**
84) **9/2 + 1/12**
85) **Arrange in ascending order 11/10 , 15/2 , 8/3**
86) **6 girls can build a road in 7 days. How many days will 18 girls build it?**
87) **1 oranges costs Rs 10 . How many oranges can you buy for Rs 70?**
88) **Find the square root of 7 - sqrt(48)**
89) **Multiply (-1a+3b) (+5a-1b)**
90) **Factorise -8aa -14ab -5bb**
91) **The volume of 10 Kg of material M1 is 12 litres, and the volume of 13 KG of material M2 is 8 litres. Which material is heavier?**
92) **The molecular mass of a compound C is 18. How many atoms are there in 6 grams of the compound?**
93) **The atomic number of an element E is 4, and its mass number is 21. How many neutrons does the element have?**
94) **Universal set = (q, k, p, f, n, o, c, l, e, t) set A = (k, f, l, e, c), set B = (t, q, o, k, e, n) . Find A union B by drawing venn diagrams**
95) **Factorize 8*v*v*v + 1*t*t*t**
96) **If you have scored 19 marks in History out of 170 marks, what is the percentage of marks**

scored by you
97) **The angles of a triangle are in the ratio of 1, 4 and 4. Find their absolute values**
98) **According to Newton's second law, force is proportional to**
99) **The initial velocity of a ball of mass 4 kg is 13 metre/s and the final velocity is 8 metre/sec. What is the change in velocity**
100) **The initial velocity of a ball of mass 2 kg is 9 metre/s and the final velocity is 7 metre/sec. What is the change in momentum?**
101) **The initial velocity of a ball of mass 4 kg is 2 metre/s, and after 7 secs the final velocity is 8 metre/sec. What is the rate of change in momentum?**
102) **A 6 kg rifle fires a 10 g bullet at a velocity of 15 m/s find the recoil velocity of the rifle?**
103) **A 13 g bullet at a travelling at a velocity of 15 m/s hits a stationary plank of wood of mass 11 g and gets embedded in the plank. What is the velocity with which the combined mass of the wood and the bullet moves ?**
104) **An airplane accelerates down a runway at 12 m/s*s for 4 s until it finally lifts off the ground. Determine the distance traveled before takeoff**
105) **In order to gain a velocity of 4 m/s how long should a force of 6 N be exerted on a body of mass 12 kg that is initially at rest?**
106) **Radius of a circle is 56 cm. Find its area**
107) **What is the distance between the points (-2,3) and (-3, -1)?**
108) **Find the coordinate of the point that divides the points (-11,5) and (-5, 3) internally in the ratio of 16:20?**
109) **What is the area of a right angled triangle with base = 18 cm and height = 5 cm ?**
110) **What is the area of a isosceles triangle with base = 15 cm and height = 16 cm ?**
111) **What is the radius of a circle with a chord = 9 cm and distance of the chord from centre = 19? cm**
112) **What number added to -14 gives 26?**
113) **What number multiplied by -16 gives 27?**
114) **What number divided by -22 gives -4?**
115) **Amrita and Pooja have some money. If you multiply Amrita 's money by 4 and Pooja 's money by 11 and add them up, you get Rs 140. If you multiply Amrita 's money by 14 and Pooja 's money by 6 and add them up, you get Rs 100. How much money does each person have?**
116) **A father is 6 times as old as his son, after 10 years, the father will be 4.1 as old as his son at that time, therefore, the fathers current age is:**
117) **Two numbers are in the ratio 6. If you add 12 to both, their ratio becomes 3.4. Find the numbers :**
118) **One piece of pipe 31 meters long is to be cut into two pieces, with the lengths of the pieces being in a 15 : 14 ratio. What are the lengths of the pieces?**
119) **Find the unknown value in the proportion: 3 : x = 11 : 1.**
120) **x is directly proportional to y. When x is -18, y is 11. Find x when y is -6.**

ANSWERS id=24888 3
=================================
1) **-25**
2) **15**
3) **0.68**
4) **4.9**
5) **1/11, 9/6, 8/5**
6) **1.38**
7) **77**
8) **sqrt(9) - sqrt(2), or sqrt(2) - sqrt(9)**
9) **+8aa -4ab -4bb**
10) **(-4a+4b) (+1a+4b)**
11) **M1**
12) **18/5 * 6.023 *** 10^{23}
13) **3**
14) **(o,j,m)**
15) **(5g + 2b) (25g*g - 10gb + 4b*b)**
16) **0**
17) **80 85 15**
18) **rate of change of momentum**
19) **5 metre/sec**
20) **18 (kg*metre)/sec**
21) **8.0 metre/(sec*sec)**
22) **7.6**
23) **120.0**
24) **112**
25) **67.5**
26) **5544**
27) **sqrt (53.000)**
28) **(10.6, -1.7)**
29) **17.5**
30) **6**
31) **18.4**
32) **58**
33) **-4.6**
34) **588**
35) **Rishi = 9, Pooja = 13**
36) **64**
37) **120**
38) **19.094 and 19.094**
39) **3.421**
40) **-8.400**
ANSWERS id=24888 2
=================================
41) **-18**
42) **10**
43) **0.07**
44) **2.92**
45) **5/14, 15/9, 11/4**
46) **90**
47) **77**

48) **sqrt(9) + sqrt(4)**
49) **-6aa +3ab +3bb**
50) **(-1a-4b) (+5a-4b)**
51) **M2**
52) **20/24 * 6.023 *** 10^{23}
53) **8**
54) **(u,l,h)**
55) **(1t + 2v) (1t*t - 2tv + 4v*v)**
56) **0**
57) **30 45 105**
58) **rate of change of momentum**
59) **-10 metre/sec**
60) **1 (kg*metre)/sec**
61) **10.0 metre/(sec*sec)**
62) **16.5**
63) **16.2**
64) **45**
65) **52.5**
66) **3850**
67) **sqrt (145.000)**
68) **(0.1, -7.8)**
69) **39**
70) **195**
71) **21.5**
72) **-62**
73) **-10.3333333333333**
74) **-1900**
75) **Amba = 11, Ravi = 8**
76) **90**
77) **84**
78) **34.696 and 34.696**
79) **11.875**
80) **-85.500**
ANSWERS id=24888 1
================================
81) **-13**
82) **14**
83) **0.36**
84) **4.58**
85) **11/10, 8/3, 15/2**
86) **2.33**
87) **7**
88) **sqrt(3) - sqrt(4), or sqrt(4) - sqrt(3)**
89) **-5aa +16ab -3bb**
90) **(-2a-1b) (+4a+5b)**
91) **M2**
92) **6/18 * 6.023 *** 10^{23}
93) **17**
94) **(e,n,l,c,k,q,f,t,o)**
95) **(2v + 1t) (4v*v - 2vt + 1t*t)**
96) **0**

97) **20 80 80**
98) **rate of change of momentum**
99) **-5 metre/sec**
100) **-4 (kg*metre)/sec**
101) **3.4 metre/(sec*sec)**
102) **25.0**
103) **17.7**
104) **96**
105) **8.0**
106) **9856**
107) **sqrt (17.000)**
108) **(-8.3, 4.1)**
109) **45**
110) **240**
111) **21.0**
112) **40**
113) **-1.6875**
114) **88**
115) **Amrita = 2, Pooja = 12**
116) **96**
117) **66**
118) **16.034 and 16.034**
119) **0.273**
120) **3.667**
