# Supplementary material for: Sequence/structural analysis of xylem proteome emphasizes pathogenesis-related proteins, chitinases and β-1, 3-glucanases as key players in grapevine defense against Xylella fastidiosa
Source: PeerJ. 2016 May 24;4:e2007. doi: 10.7717/peerj.2007 (PMC4888286; doi:10.7717/peerj.2007)
Supplement: File S3 [file peerj-04-2007-s005.zip › perl_scripts/QP/FigQpaper/Class9/doc1.Sept2013.pdf]

Template

Sandeep Chakraborty[1],
1. Department of Biological Sciences, Tata Institute of Fundamental Research, Homi Bhabha Road, Mumbai 400 005, India.

Abstract

Introduction

1 Question set

1) **15 men can build a garden in 12 days. How many days will 165 men build it?**
2) **The atomic number of an element E is 8, and its mass number is 11. How many neutrons does the element have?**
3) **Amrita and Amit have some money. If you multiply Amrita 's money by 12 and Amit 's money by 6 and add them up, you get Rs 246. If you multiply Amrita 's money by 1 and Amit 's money by 8 and add them up, you get Rs 103. How much money does each person have?**
4) **Factorize** $8t^3 - 64o^3$
5) **Find the square root of 4 - sqrt(12)**

2 Question set

1) **Arrange in ascending order 3/15 , 7/6 , 12/11**
2) **Factorise -16ab -16bb -4aa**
3) **13 men can build a house in 9 days. How many men will make it in 81 days ?**
4) **Find the square root of 9 + sqrt(32)**
5) **1/10+5/15**

3 Question set

1) **8/12+10/6**
2) **The molecular mass of a compound C is 11. How many atoms are there in 15 grams of the compound?**
3) **Arrange in descending order 13/12 , 14/3 , 1/5**
4) **Find the square root of 12 - sqrt(128)**
5) **Universal set = (z, m, c, a, t, q, s, w, e, b) set A = (b, e, m, a, z), set B = (m, a, w, q, e, s) . Find A union B by drawing venn diagrams**

4 Question set

1) **Universal set = (v, q, y, e, m, h, p, f, o, k) set A = (e, o, y, m, f), set B = (q, m, y, p, v, h) . Find A intersection B by drawing venn diagrams**
2) **The volume of 15 Kg of material M1 is 6 litres, and the volume of 3 KG of material M2 is 13 litres. Which material is heavier?**
3) **Arrange in descending order 12/3 , 7/4 , 8/11**
4) **Find the square root of 6 - sqrt(32)**
5) **Find the sum of: - 20 + 16 + 5 - 18 + 3**

5 Question set

1) **The atomic number of an element E is 5, and its mass number is 25. How many neutrons does the element have?**
2) **Factorise -4ab -16bb +12aa**

3) **12 boys can build a house in 14 days. How many boys will make it in 112 days ?**
4) **multiply (-1a-4b) (-2a-1b)**
5) **The molecular mass of a compound C is 16. How many atoms are there in 9 grams of the compound?**

6 Question set

1) **10/8+4/11**
2) **1 books costs Rs 12 . What is the cost of 3 books?**
3) **Factorise -19ab +3bb +20aa**
4) **The atomic number of an element E is 7, and its mass number is 22. How many neutrons does the element have?**
5) **5 boys can build a bridge in 3 days. How many boys will make it in 6 days ?**

7 Question set

1) **Factorize** $64r^3 - 1c^3$
2) **Radius of a circle is 49 cm. Find its area and circumference**
3) **The volume of 9 Kg of material M1 is 4 litres, and the volume of 17 KG of material M2 is 2 litres. Which material is heavier?**
4) **Factorise +2ab +15bb -8aa**
5) **Find the sum of: - 19 - 6 - 11 + 6 - 17**

8 Question set

1) **multiply (+2a+3b) (+1a+5b)**
2) **17/14+3/8**
3) **The volume of 20 Kg of material M1 is 11 litres, and the volume of 7 KG of material M2 is 3 litres. Which material is heavier?**
4) **Rishi and Pooja have some money. If you multiply Rishi 's money by 1 and Pooja 's money by 13 and add them up, you get Rs 87. If you multiply Rishi 's money by 10 and Pooja 's money by 14 and add them up, you get Rs 174. How much money does each person have?**
5) **The molecular mass of a compound C is 30. How many atoms are there in 11 grams of the compound?**

9 Question set

1) **Find the sum of: - 20 + 15 - 9 - 19 - 17**
2) **The atomic number of an element E is 4, and its mass number is 28. How many neutrons does the element have?**
3) **9/2+13/16**
4) **Universal set = (d, p, r, b, m, t, l, i, y, n) set A = (i, r, b, l, y), set B = (n, b, y, r, d, l) . Find A intersection B by drawing venn diagrams**
5) **Amit and Ravi have some money. If you multiply Amit 's money by 15 and Ravi 's money**

by 5 and add them up, you get Rs 250. If you multiply Amit 's money by 11 and Ravi 's money by 7 and add them up, you get Rs 230. How much money does each person have?

10 Question set

1) **The volume of 17 Kg of material M1 is 12 litres, and the volume of 13 KG of material M2 is 14 litres. Which material is heavier?**
2) **Radius of a circle is 7 cm. Find its area and circumference**
3) **Find the square root of 10 + sqrt(84)**
4) **Arrange in descending order 3/1 , 11/15 , 10/5**
5) **Find the sum of: + 4 - 12 + 2 - 8 + 9**

11 Question set

1) **Factorise +4ab +4bb -8aa**
2) **Universal set = (j, r, k, t, w, d, c, h, v, a) set A = (j, c, h, a, d), set B = (k, j, t, h, r, c) . Find A intersection B by drawing venn diagrams**
3) **Find the sum of: - 6 - 14 - 17 - 1 + 2**
4) **The volume of 6 Kg of material M1 is 5 litres, and the volume of 10 KG of material M2 is 9 litres. Which material is heavier?**
5) **8 women can build a house in 5 days. How many women will make it in 55 days ?**

12 Question set

1) **Radius of a circle is 56 cm. Find its area and circumference**
2) **Factorize** $1i^3 + 27a^3$
3) **Find the sum of: + 3 + 15 - 16 - 18 + 2**
4) **Sanjay and Amrita have some money. If you multiply Sanjay 's money by 6 and Amrita 's money by 2 and add them up, you get Rs 62. If you multiply Sanjay 's money by 5 and Amrita 's money by 1 and add them up, you get Rs 51. How much money does each person have?**
5) **The molecular mass of a compound C is 28. How many atoms are there in 19 grams of the compound?**

13 Question set

1) **multiply (-2a-1b) (+4a+1b)**
2) **Find the sum of: - 13 + 4 - 16 - 9 - 3**
3) **Factorise +0ab -10bb +10aa**
4) **The atomic number of an element E is 6, and its mass number is 14. How many neutrons does the element have?**
5) **Arrange in ascending order 13/10 , 2/12 , 1/6**

14 Question set

1) **18/10+1/12**
2) **Find the square root of 8 + sqrt(28)**
3) **Radius of a circle is 14 cm. Find its area and circumference**
4) **Find the sum of: + 17 - 19 - 14 + 11 + 1**
5) **multiply (-2a-2b) (+1a+4b)**

15 Question set

1) **multiply (+4a-1b) (+2a+4b)**
2) **13 apples costs Rs 2 . How many apples can you buy for Rs 20?**
3) **Find the square root of 8 + sqrt(60)**
4) **Radius of a circle is 14 cm. Find its area and circumference**
5) **Find the sum of: - 3 + 8 - 8 - 10 - 19**

16 Question set

1) **Universal set = (n, c, e, l, p, x, k, g, y, s) set A = (g, l, y, c, p), set B = (s, k, g, n, p, c) . Find A intersection B by drawing venn diagrams**
2) **6 girls can build a house in 10 days. How many girls will make it in 20 days ?**
3) **Find the sum of: - 11 + 5 + 19 - 10 - 5**
4) **6 books costs Rs 1 . What is the cost of 24 books?**
5) **multiply (-2a-5b) (-1a+3b)**

17 Question set

1) **The volume of 4 Kg of material M1 is 6 litres, and the volume of 9 KG of material M2 is 10 litres. Which material is heavier?**
2) **Find the square root of 11 - sqrt(96)**
3) **2 girls can build a bridge in 3 days. How many girls will make it in 15 days ?**
4) **Universal set = (w, m, t, j, u, b, n, a, o, i) set A = (n, t, b, a, u), set B = (j, o, w, n, u, m) . Find A intersection B by drawing venn diagrams**
5) **multiply (+4a+3b) (+5a-1b)**

18 Question set

1) **Universal set = (y, u, v, p, h, r, s, d, j, t) set A = (h, s, v, r, u), set B = (p, d, h, j, r, t) . Find A union B by drawing venn diagrams**
2) **The volume of 10 Kg of material M1 is 8 litres, and the volume of 1 KG of material M2 is 12 litres. Which material is heavier?**
3) **6/17+13/3**
4) **Rishi and Amba have some money. If you multiply Rishi 's money by 10 and Amba 's money by 7 and add them up, you get Rs 140. If you multiply Rishi 's money by 15 and Amba 's money by 8 and add them up, you get Rs 185. How much money does each person have?**

5) **Find the sum of: + 13 - 18 - 18 - 12 + 8**

19 Question set

1) **Universal set = (h, e, a, r, o, x, l, g, p, v) set A = (p, h, e, x, a), set B = (r, h, g, a, o, e) . Find A intersection B by drawing venn diagrams**
2) **Arrange in ascending order 5/6 , 14/12 , 1/7**
3) **The atomic number of an element E is 7, and its mass number is 24. How many neutrons does the element have?**
4) **Find the square root of 11 - sqrt(96)**
5) **Factorise +12ab +4bb +8aa**

20 Question set

1) **Factorize** $64u^3 + 125g^3$
2) **Universal set = (d, r, q, m, s, g, u, w, v, b) set A = (r, s, q, v, m), set B = (r, u, w, s, d, v) . Find A union B by drawing venn diagrams**
3) **8/14+15/18**
4) **The atomic number of an element E is 4, and its mass number is 14. How many neutrons does the element have?**
5) **12 books costs Rs 15 . What is the cost of 72 books?**

21 Question set

1) **Find the square root of 17 - sqrt(280)**
2) **10 men can build a house in 5 days. How many men will make it in 30 days ?**
3) **The atomic number of an element E is 24, and its mass number is 27. How many neutrons does the element have?**
4) **The volume of 9 Kg of material M1 is 13 litres, and the volume of 20 KG of material M2 is 2 litres. Which material is heavier?**
5) **The molecular mass of a compound C is 22. How many atoms are there in 10 grams of the compound?**

22 Question set

1) **multiply (-2a+1b) (+5a-3b)**
2) **14 men can build a road in 7 days. How many days will 70 men build it?**
3) **14 mangoes costs Rs 5 . How many mangoes can you buy for Rs 25?**
4) **Universal set = (p, x, l, g, v, w, b, i, d, a) set A = (w, d, x, g, b), set B = (d, p, w, i, a, v) . Find A union B by drawing venn diagrams**
5) **Factorize** $27w^3 - 1v^3$

23 Question set

1) **The molecular mass of a compound C is 30. How many atoms are there in 11 grams of the compound?**
2) **Factorise +0ab -20bb +20aa**
3) **Find the sum of: - 3 + 1 + 3 - 2 - 13**
4) **Arrange in ascending order 11/13 , 6/12 , 3/8**
5) **Ritu and Ravi have some money. If you multiply Ritu 's money by 15 and Ravi 's money by 5 and add them up, you get Rs 125. If you multiply Ritu 's money by 9 and Ravi 's money by 11 and add them up, you get Rs 155. How much money does each person have?**

24 Question set

1) **Universal set = (f, c, u, w, n, x, k, i, m, y) set A = (c, u, m, w, n), set B = (f, y, k, m, c, x) . Find A union B by drawing venn diagrams**
2) **Factorise -2ab +4bb -20aa**
3) **Factorize** $125z^3 + 8j^3$
4) **Find the square root of 18 - sqrt(320)**
5) **Amit and Ritu have some money. If you multiply Amit 's money by 2 and Ritu 's money by 11 and add them up, you get Rs 72. If you multiply Amit 's money by 13 and Ritu 's money by 9 and add them up, you get Rs 93. How much money does each person have?**

25 Question set

1) **8 books costs Rs 9 . What is the cost of 56 books?**
2) **Factorize** $64t^3 + 125x^3$
3) **9/4+5/11**
4) **Find the sum of: - 19 - 3 - 2 - 13 - 7**
5) **Radius of a circle is 63 cm. Find its area and circumference**

26 Question set

1) **6 apples costs Rs 11 . How many apples can you buy for Rs 33?**
2) **Shalini and Ritu have some money. If you multiply Shalini 's money by 2 and Ritu 's money by 10 and add them up, you get Rs 52. If you multiply Shalini 's money by 3 and Ritu 's money by 14 and add them up, you get Rs 73. How much money does each person have?**
3) **Factorise +20ab +25bb +4aa**
4) **multiply (+1a-3b) (+5a+3b)**
5) **Arrange in ascending order 13/15 , 6/3 , 8/12**

27 Question set

1) **Universal set = (q, j, l, b, c, n, i, u, t, y) set A = (u, t, y, q, l), set B = (t, i, c, u, n, q) . Find A union B by drawing venn diagrams**
2) **6 men can build a house in 4 days. How many days will 66 men build it?**

3) **Factorize** $125y^3 - 216s^3$
4) **Find the sum of: - 18 + 7 + 8 + 8 - 13**
5) **The volume of 17 Kg of material M1 is 5 litres, and the volume of 2 KG of material M2 is 6 litres. Which material is heavier?**

ANSWERS id=12412 1
==================================
1) **15 men can build a garden in 12 days. How many days will 165 men build it? = 1.09**
2) **The atomic number of an element E is 8, and its mass number is 11. How many neutrons does the element have? = 3**
3) **Amrita and Amit have some money. If you multiply Amrita 's money by 12 and Amit 's money by 6 and add them up, you get Rs 246. If you multiply Amrita 's money by 1 and Amit 's money by 8 and add them up, you get Rs 103. How much money does each person have? = Amrita = 15, Amit = 11**
4) **Factorize** $8t^3 - 64o^3 = (2t + 4o)(4t^2 - 8to + 16o^2)$
5) **Find the square root of 4 - sqrt(12) = sqrt(3) - sqrt(1), or sqrt(1) - sqrt(3)**
ANSWERS id=13295 1
==================================
1) **3/15 12/11 7/6**
2) **-16ab -16bb -4aa = (+2a+4b) (-2a-4b)**
3) **13 men can build a house in 9 days. How many men will make it in 81 days ? = 1.44**
4) **Find the square root of 9 + sqrt(32) = sqrt(8) + sqrt(1)**
5) **1/10+5/15 = 0.43**
ANSWERS id=13651 1
==================================
1) **8/12+10/6 = 2.33**
2) **The molecular mass of a compound C is 11. How many atoms are there in 15 grams of the compound? = 15/11 * 6.023 *** 10^{23}
3) **14/3 1/5 13/12**
4) **Find the square root of 12 - sqrt(128) = sqrt(8) - sqrt(4), or sqrt(4) - sqrt(8)**
5) **Universal set = (z, m, c, a, t, q, s, w, e, b) set A = (b, e, m, a, z), set B = (m, a, w, q, e, s) . Find A union B by drawing venn diagrams = (w,e,a,m,s,b,q,z)**
ANSWERS id=18718 1
==================================
1) **Universal set = (v, q, y, e, m, h, p, f, o, k) set A = (e, o, y, m, f), set B = (q, m, y, p, v, h) . Find A intersection B by drawing venn diagrams = (m,y)**
2) **The volume of 15 Kg of material M1 is 6 litres, and the volume of 3 KG of material M2 is 13 litres. Which material is heavier? = M1**
3) **12/3 8/11 7/4**
4) **Find the square root of 6 - sqrt(32) = sqrt(4) - sqrt(2), or sqrt(2) - sqrt(4)**
5) **- 20 + 16 + 5 - 18 + 3 = -14**
ANSWERS id=22159 1
==================================
1) **The atomic number of an element E is 5, and its mass number is 25. How many neutrons does the element have? = 20**
2) **-4ab -16bb +12aa = (+3a-4b) (+4a+4b)**
3) **12 boys can build a house in 14 days. How many boys will make it in 112 days ? = 1.5**
4) **(-1a-4b) (-2a-1b) = +9ab +4bb +2aa**
5) **The molecular mass of a compound C is 16. How many atoms are there in 9 grams of the compound? = 9/16 * 6.023 *** 10^{23}
ANSWERS id=2470 1
==================================
1) **10/8+4/11 = 1.61**
2) **1 books costs Rs 12 . What is the cost of 3 books? = 36**
3) **-19ab +3bb +20aa = (+4a-3b) (+5a-1b)**
4) **The atomic number of an element E is 7, and its mass number is 22. How many neutrons**

does the element have? = 15
5) **5 boys can build a bridge in 3 days. How many boys will make it in 6 days ? = 2.5**
ANSWERS id=28808 1
==================================
1) **Factorize** $64r^3 - 1c^3 = (4r + 1c)(16r^2 - 4rc + 1c^2)$
2) **Area = 7539.14 sq cm , Circumference = 307.72 cm**
3) **The volume of 9 Kg of material M1 is 4 litres, and the volume of 17 KG of material M2 is 2 litres. Which material is heavier? =** M2
4) **+2ab +15bb -8aa = (+2a-3b) (-4a-5b)**
5) **- 19 - 6 - 11 + 6 - 17 = -47**
ANSWERS id=31173 1
==================================
1) **(+2a+3b) (+1a+5b) = +13ab +15bb +2aa**
2) **17/14+3/8 = 1.59**
3) **The volume of 20 Kg of material M1 is 11 litres, and the volume of 7 KG of material M2 is 3 litres. Which material is heavier? =** M2
4) **Rishi and Pooja have some money. If you multiply Rishi 's money by 1 and Pooja 's money by 13 and add them up, you get Rs 87. If you multiply Rishi 's money by 10 and Pooja 's money by 14 and add them up, you get Rs 174. How much money does each person have? = Rishi = 9, Pooja = 6**
5) **The molecular mass of a compound C is 30. How many atoms are there in 11 grams of the compound? = 11/30 * 6.023 *** 10^{23}
ANSWERS id=31994 1
==================================
1) **- 20 + 15 - 9 - 19 - 17 = -50**
2) **The atomic number of an element E is 4, and its mass number is 28. How many neutrons does the element have? = 24**
3) **9/2+13/16 = 5.31**
4) **Universal set = (d, p, r, b, m, t, l, i, y, n) set A = (i, r, b, l, y), set B = (n, b, y, r, d, l) . Find A intersection B by drawing venn diagrams = (b,y,r,l)**
5) **Amit and Ravi have some money. If you multiply Amit 's money by 15 and Ravi 's money by 5 and add them up, you get Rs 250. If you multiply Amit 's money by 11 and Ravi 's money by 7 and add them up, you get Rs 230. How much money does each person have? = Amit = 12, Ravi = 14**
ANSWERS id=35189 1
==================================
1) **The volume of 17 Kg of material M1 is 12 litres, and the volume of 13 KG of material M2 is 14 litres. Which material is heavier? =** M1
2) **Area = 153.86 sq cm , Circumference = 43.96 cm**
3) **Find the square root of 10 + sqrt(84) = sqrt(7) + sqrt(3)**
4) **3/1 10/5 11/15**
5) **+ 4 - 12 + 2 - 8 + 9 = -5**
ANSWERS id=39982 1
==================================
1) **+4ab +4bb -8aa = (-2a-1b) (+4a-4b)**
2) **Universal set = (j, r, k, t, w, d, c, h, v, a) set A = (j, c, h, a, d), set B = (k, j, t, h, r, c) . Find A intersection B by drawing venn diagrams = (j,h,c)**
3) **- 6 - 14 - 17 - 1 + 2 = -36**
4) **The volume of 6 Kg of material M1 is 5 litres, and the volume of 10 KG of material M2 is 9 litres. Which material is heavier? =** M1
5) **8 women can build a house in 5 days. How many women will make it in 55 days ? = 0.73**

ANSWERS id=5050 1
==================================
1) **Area = 9847.04 sq cm , Circumference = 351.68 cm**
2) **Factorize** $1i^3 + 27a^3 = (1i - 3a)(1i^2 + 3ia + 9a^2)$
3) **+ 3 + 15 - 16 - 18 + 2 = -14**
4) **Sanjay and Amrita have some money. If you multiply Sanjay 's money by 6 and Amrita 's money by 2 and add them up, you get Rs 62. If you multiply Sanjay 's money by 5 and Amrita 's money by 1 and add them up, you get Rs 51. How much money does each person have? = Sanjay = 10, Amrita = 1**
5) **The molecular mass of a compound C is 28. How many atoms are there in 19 grams of the compound? = 19/28 * 6.023 *** 10^{23}
ANSWERS id=58236 1
==================================
1) **(-2a-1b) (+4a+1b) = -6ab -1bb -8aa**
2) **- 13 + 4 - 16 - 9 - 3 = -37**
3) **+0ab -10bb +10aa = (+2a-2b) (+5a+5b)**
4) **The atomic number of an element E is 6, and its mass number is 14. How many neutrons does the element have? = 8**
5) **2/12 1/6 13/10**
ANSWERS id=62840 1
==================================
1) **18/10+1/12 = 1.88**
2) **Find the square root of 8 + sqrt(28) = sqrt(7) + sqrt(1)**
3) **Area = 615.44 sq cm , Circumference = 87.92 cm**
4) **+ 17 - 19 - 14 + 11 + 1 = -4**
5) **(-2a-2b) (+1a+4b) = -10ab -8bb -2aa**
ANSWERS id=64643 1
==================================
1) **(+4a-1b) (+2a+4b) = +14ab -4bb +8aa**
2) **13 apples costs Rs 2 . How many apples can you buy for Rs 20? = 130**
3) **Find the square root of 8 + sqrt(60) = sqrt(5) + sqrt(3)**
4) **Area = 615.44 sq cm , Circumference = 87.92 cm**
5) **- 3 + 8 - 8 - 10 - 19 = -32**
ANSWERS id=65612 1
==================================
1) **Universal set = (n, c, e, l, p, x, k, g, y, s) set A = (g, l, y, c, p), set B = (s, k, g, n, p, c) . Find A intersection B by drawing venn diagrams = (g,p,c)**
2) **6 girls can build a house in 10 days. How many girls will make it in 20 days ? = 3**
3) **- 11 + 5 + 19 - 10 - 5 = -2**
4) **6 books costs Rs 1 . What is the cost of 24 books? = 4**
5) **(-2a-5b) (-1a+3b) = -1ab -15bb +2aa**
ANSWERS id=65736 1
==================================
1) **The volume of 4 Kg of material M1 is 6 litres, and the volume of 9 KG of material M2 is 10 litres. Which material is heavier? = M2**
2) **Find the square root of 11 - sqrt(96) = sqrt(8) - sqrt(3), or sqrt(3) - sqrt(8)**
3) **2 girls can build a bridge in 3 days. How many girls will make it in 15 days ? = 0.4**
4) **Universal set = (w, m, t, j, u, b, n, a, o, i) set A = (n, t, b, a, u), set B = (j, o, w, n, u, m) . Find A intersection B by drawing venn diagrams = (n,u)**
5) **(+4a+3b) (+5a-1b) = +11ab -3bb +20aa**
ANSWERS id=67093 1

==================================

1) **Universal set = (y, u, v, p, h, r, s, d, j, t) set A = (h, s, v, r, u), set B = (p, d, h, j, r, t) . Find A union B by drawing venn diagrams = (r,d,v,s,j,u,p,h,t)**

2) **The volume of 10 Kg of material M1 is 8 litres, and the volume of 1 KG of material M2 is 12 litres. Which material is heavier? = M1**

3) **6/17+13/3 = 4.69**

4) **Rishi and Amba have some money. If you multiply Rishi 's money by 10 and Amba 's money by 7 and add them up, you get Rs 140. If you multiply Rishi 's money by 15 and Amba 's money by 8 and add them up, you get Rs 185. How much money does each person have? = Rishi = 7, Amba = 10**

5) **+ 13 - 18 - 18 - 12 + 8 = -27**

ANSWERS id=70142 1

==================================

1) **Universal set = (h, e, a, r, o, x, l, g, p, v) set A = (p, h, e, x, a), set B = (r, h, g, a, o, e) . Find A intersection B by drawing venn diagrams = (h,a,e)**

2) **5/6 1/7 14/12**

3) **The atomic number of an element E is 7, and its mass number is 24. How many neutrons does the element have? = 17**

4) **Find the square root of 11 - sqrt(96) = sqrt(3) - sqrt(8), or sqrt(8) - sqrt(3)**

5) **+12ab +4bb +8aa = (+4a+2b) (+2a+2b)**

ANSWERS id=78545 1

==================================

1) **Factorize** $64u^3 + 125g^3 = (4u - 5g)(16u^2 + 20ug + 25g^2)$

2) **Universal set = (d, r, q, m, s, g, u, w, v, b) set A = (r, s, q, v, m), set B = (r, u, w, s, d, v) . Find A union B by drawing venn diagrams = (w,r,d,m,v,s,u,q)**

3) **8/14+15/18 = 1.4**

4) **The atomic number of an element E is 4, and its mass number is 14. How many neutrons does the element have? = 10**

5) **12 books costs Rs 15 . What is the cost of 72 books? = 90**

ANSWERS id=88069 1

==================================

1) **Find the square root of 17 - sqrt(280) = sqrt(7) - sqrt(10), or sqrt(10) - sqrt(7)**

2) **10 men can build a house in 5 days. How many men will make it in 30 days ? = 1.67**

3) **The atomic number of an element E is 24, and its mass number is 27. How many neutrons does the element have? = 3**

4) **The volume of 9 Kg of material M1 is 13 litres, and the volume of 20 KG of material M2 is 2 litres. Which material is heavier? = M2**

5) **The molecular mass of a compound C is 22. How many atoms are there in 10 grams of the compound? = 10/22 * 6.023 *** 10^{23}

ANSWERS id=89080 1

==================================

1) **(-2a+1b) (+5a-3b) = +11ab -3bb -10aa**

2) **14 men can build a road in 7 days. How many days will 70 men build it? = 1.4**

3) **14 mangoes costs Rs 5 . How many mangoes can you buy for Rs 25? = 70**

4) **Universal set = (p, x, l, g, v, w, b, i, d, a) set A = (w, d, x, g, b), set B = (d, p, w, i, a, v) . Find A union B by drawing venn diagrams = (w,a,v,x,d,p,g,b,i)**

5) **Factorize** $27w^3 - 1v^3 = (3w + 1v)(9w^2 - 3wv + 1v^2)$

ANSWERS id=90568 1

==================================

1) **The molecular mass of a compound C is 30. How many atoms are there in 11 grams of the compound? = 11/30 * 6.023 *** 10^{23}

2) **+0ab -20bb +20aa = (+4a+4b) (+5a-5b)**
3) **- 3 + 1 + 3 - 2 - 13 = -14**
4) **6/12 3/8 11/13**
5) **Ritu and Ravi have some money. If you multiply Ritu 's money by 15 and Ravi 's money by 5 and add them up, you get Rs 125. If you multiply Ritu 's money by 9 and Ravi 's money by 11 and add them up, you get Rs 155. How much money does each person have? = Ritu = 5, Ravi = 10**
ANSWERS id=94507 1
==============================
1) **Universal set = (f, c, u, w, n, x, k, i, m, y) set A = (c, u, m, w, n), set B = (f, y, k, m, c, x) . Find A union B by drawing venn diagrams = (w,n,x,m,y,c,u,k,f)**
2) **-2ab +4bb -20aa = (-5a+2b) (+4a+2b)**
3) **Factorize** $125z^3 + 8j^3 = (5z - 2j)(25z^2 + 10zj + 4j^2)$
4) **Find the square root of 18 - sqrt(320) = sqrt(10) - sqrt(8), or sqrt(8) - sqrt(10)**
5) **Amit and Ritu have some money. If you multiply Amit 's money by 2 and Ritu 's money by 11 and add them up, you get Rs 72. If you multiply Amit 's money by 13 and Ritu 's money by 9 and add them up, you get Rs 93. How much money does each person have? = Amit = 3, Ritu = 6**
ANSWERS id=94760 1
==============================
1) **8 books costs Rs 9 . What is the cost of 56 books? = 63**
2) **Factorize** $64t^3 + 125x^3 = (4t - 5x)(16t^2 + 20tx + 25x^2)$
3) **9/4+5/11 = 2.7**
4) **- 19 - 3 - 2 - 13 - 7 = -44**
5) **Area = 12462.66 sq cm , Circumference = 395.64 cm**
ANSWERS id=96256 1
==============================
1) **6 apples costs Rs 11 . How many apples can you buy for Rs 33? = 18**
2) **Shalini and Ritu have some money. If you multiply Shalini 's money by 2 and Ritu 's money by 10 and add them up, you get Rs 52. If you multiply Shalini 's money by 3 and Ritu 's money by 14 and add them up, you get Rs 73. How much money does each person have? = Shalini = 1, Ritu = 5**
3) **+20ab +25bb +4aa = (+2a+5b) (+2a+5b)**
4) **(+1a-3b) (+5a+3b) = -12ab -9bb +5aa**
5) **13/15 8/12 6/3**
ANSWERS id=97051 1
==============================
1) **Universal set = (q, j, l, b, c, n, i, u, t, y) set A = (u, t, y, q, l), set B = (t, i, c, u, n, q) . Find A union B by drawing venn diagrams = (n,y,l,u,c,q,t,i)**
2) **6 men can build a house in 4 days. How many days will 66 men build it? = 0.36**
3) **Factorize** $125y^3 - 216s^3 = (5y + 6s)(25y^2 - 30ys + 36s^2)$
4) **- 18 + 7 + 8 + 8 - 13 = -8**
5) **The volume of 17 Kg of material M1 is 5 litres, and the volume of 2 KG of material M2 is 6 litres. Which material is heavier? = M1**
