# Supplementary material for: Sequence/structural analysis of xylem proteome emphasizes pathogenesis-related proteins, chitinases and β-1, 3-glucanases as key players in grapevine defense against Xylella fastidiosa
Source: PeerJ. 2016 May 24;4:e2007. doi: 10.7717/peerj.2007 (PMC4888286; doi:10.7717/peerj.2007)
Supplement: File S3 [file peerj-04-2007-s005.zip › perl_scripts/QP/FigQpaper/16thJuly/Q.pdf]

QUESTIONS id=32679 1
==================================

1) **Find the sum of: - 15 - 10 - 12 + 11 - 4 - 11 + 8 - 8**
2) **5/8+11/6**
3) **Radius of a circle is 39 cm. Find its area and circumference**
4) **5 girls can build a house in 22 days. How many days will 25 girls build it?**
5) **30 mangoes costs Rs 41 . How many mangoes can you buy for Rs 492?**
6) **Arrange in descending order 3/9 , 14/4 , 15/15**
7) **Find the square root of 5 + sqrt(24)**
8) **multiply (-7a+5b) (-5a+2b)**
9) **Factorise +96ab -54bb -42aa**

QUESTIONS id=36669 1
==================================

1) **Find the sum of: - 12 + 17 - 9 + 7 + 4 - 1 + 19 + 15**
2) **9/19+11/8**
3) **Radius of a circle is 34 cm. Find its area and circumference**
4) **25 women can build a bridge in 15 days. How many days will 700 women build it?**
5) **8 oranges costs Rs 14 . How many oranges can you buy for Rs 182?**
6) **Arrange in descending order 11/16 , 11/11 , 17/1**
7) **Find the square root of 10 + sqrt(100)**
8) **multiply (-3a+2b) (-4a-2b)**
9) **Factorise +42ab -27bb -16aa**

QUESTIONS id=38674 1
==================================

1) **Find the sum of: - 17 + 13 + 3 - 17 + 9 + 3 + 8 - 6**
2) **8/14+11/11**
3) **Radius of a circle is 9 cm. Find its area and circumference**
4) **16 men can build a house in 30 days. How many days will 352 men build it?**
5) **23 mangoes costs Rs 39 . How many mangoes can you buy for Rs 429?**
6) **Arrange in descending order 7/9 , 8/1 , 20/17**
7) **Find the square root of 11 + sqrt(72)**
8) **multiply (-8a+8b) (-3a-6b)**
9) **Factorise +9ab +63bb -72aa**

QUESTIONS id=43328 1
==================================

1) **Find the sum of: + 12 + 3 - 3 + 6 - 6 + 20 + 5 + 6**
2) **5/11+20/9**
3) **Radius of a circle is 16 cm. Find its area and circumference**
4) **30 girls can build a garden in 23 days. How many days will 810 girls build it?**
5) **15 oranges costs Rs 33 . How many oranges can you buy for Rs 627?**
6) **Arrange in descending order 14/10 , 9/2 , 8/7**
7) **Find the square root of 14 + sqrt(160)**
8) **multiply (+3a+7b) (-7a-2b)**
9) **Factorise +58ab +7bb -45aa**

QUESTIONS id=57450 1
==================================

1) **Find the sum of: - 14 + 11 - 8 - 2 - 12 + 7 + 3 + 2**
2) **6/1+18/11**
3) **Radius of a circle is 22 cm. Find its area and circumference**
4) **6 women can build a bridge in 15 days. How many women will make it in 330 days ?**
5) **1 books costs Rs 12 . What is the cost of 9 books?**

6) **Arrange in ascending order 9/2 , 1/14 , 5/4**
7) **Find the square root of 15 - sqrt(224)**
8) **multiply (+7a+5b) (+10a-7b)**
9) **Factorise +50ab -8bb -12aa**
QUESTIONS id=61526 1
==============================
1) **Find the sum of: + 18 - 14 - 13 + 10 + 18 - 2 + 5 + 10**
2) **8/3+13/8**
3) **Radius of a circle is 32 cm. Find its area and circumference**
4) **6 men can build a road in 2 days. How many days will 42 men build it?**
5) **44 oranges costs Rs 38 . How many oranges can you buy for Rs 722?**
6) **Arrange in ascending order 18/10 , 13/7 , 16/8**
7) **Find the square root of 14 - sqrt(192)**
8) **multiply (-7a-1b) (-10a+4b)**
9) **Factorise +32ab +24bb -56aa**
QUESTIONS id=79872 1
==============================
1) **Find the sum of: + 5 - 11 - 6 - 7 + 20 - 15 + 2 - 12**
2) **4/2+18/17**
3) **Radius of a circle is 31 cm. Find its area and circumference**
4) **7 men can build a bridge in 5 days. How many men will make it in 90 days ?**
5) **40 books costs Rs 47 . What is the cost of 800 books?**
6) **Arrange in descending order 15/20 , 17/12 , 10/2**
7) **Find the square root of 12 + sqrt(144)**
8) **multiply (-7a+8b) (+3a-1b)**
9) **Factorise +32ab +16bb +16aa**
QUESTIONS id=8237 1
==============================
1) **Find the sum of: + 11 - 4 - 15 - 20 + 9 - 11 + 12 + 3**
2) **4/19+17/10**
3) **Radius of a circle is 30 cm. Find its area and circumference**
4) **11 girls can build a house in 6 days. How many girls will make it in 156 days ?**
5) **38 apples costs Rs 30 . What is the cost of 418 apples?**
6) **Arrange in ascending order 11/19 , 8/13 , 15/14**
7) **Find the square root of 8 - sqrt(60)**
8) **multiply (-3a+7b) (+2a-8b)**
9) **Factorise -16ab -3bb +12aa**
QUESTIONS id=93850 1
==============================
1) **Find the sum of: + 7 - 16 - 3 + 16 - 4 + 18 - 10 + 18**
2) **17/4+3/16**
3) **Radius of a circle is 0 cm. Find its area and circumference**
4) **24 men can build a road in 2 days. How many men will make it in 16 days ?**
5) **23 mangoes costs Rs 40 . What is the cost of 184 mangoes?**
6) **Arrange in descending order 9/12 , 11/6 , 17/18**
7) **Find the square root of 8 - sqrt(64)**
8) **multiply (-7a-7b) (-7a-9b)**
9) **Factorise -50ab -24bb -25aa**
