# Supplementary material for: Sequence/structural analysis of xylem proteome emphasizes pathogenesis-related proteins, chitinases and β-1, 3-glucanases as key players in grapevine defense against Xylella fastidiosa
Source: PeerJ. 2016 May 24;4:e2007. doi: 10.7717/peerj.2007 (PMC4888286; doi:10.7717/peerj.2007)
Supplement: File S3 [file peerj-04-2007-s005.zip › perl_scripts/QP/FigQpaper/16thJuly/A.pdf]

ANSWERS id=32679 1
==================================
1) **- 15 - 10 - 12 + 11 - 4 - 11 + 8 - 8 = -41**
2) **5/8+11/6 = 2.46**
3) **Area = 4775.94 sq cm , Circumference = 244.92 cm**
4) **5 girls can build a house in 22 days. How many days will 25 girls build it? = 4.4**
5) **30 mangoes costs Rs 41 . How many mangoes can you buy for Rs 492? = 360**
6) **14/4 15/15 3/9**
7) **Find the square root of 5 + sqrt(24) = sqrt(3) + sqrt(2)**
8) **(-7a+5b) (-5a+2b) = -39ab +10bb +35aa**
9) **+96ab -54bb -42aa = (-6a+6b) (+7a-9b)**
ANSWERS id=36669 1
==================================
1) **- 12 + 17 - 9 + 7 + 4 - 1 + 19 + 15 = 40**
2) **9/19+11/8 = 1.85**
3) **Area = 3629.84 sq cm , Circumference = 213.52 cm**
4) **25 women can build a bridge in 15 days. How many days will 700 women build it? = 0.54**
5) **8 oranges costs Rs 14 . How many oranges can you buy for Rs 182? = 104**
6) **17/1 11/11 11/16**
7) **Find the square root of 10 + sqrt(100) = sqrt(5) + sqrt(5)**
8) **(-3a+2b) (-4a-2b) = -2ab -4bb +12aa**
9) **+42ab -27bb -16aa = (+8a-9b) (-2a+3b)**
ANSWERS id=38674 1
==================================
1) **- 17 + 13 + 3 - 17 + 9 + 3 + 8 - 6 = -4**
2) **8/14+11/11 = 1.57**
3) **Area = 254.34 sq cm , Circumference = 56.52 cm**
4) **16 men can build a house in 30 days. How many days will 352 men build it? = 1.36**
5) **23 mangoes costs Rs 39 . How many mangoes can you buy for Rs 429? = 253**
6) **8/1 20/17 7/9**
7) **Find the square root of 11 + sqrt(72) = sqrt(2) + sqrt(9)**
8) **(-8a+8b) (-3a-6b) = +24ab -48bb +24aa**
9) **+9ab +63bb -72aa = (-8a-7b) (+9a-9b)**
ANSWERS id=43328 1
==================================
1) **+ 12 + 3 - 3 + 6 - 6 + 20 + 5 + 6 = 43**
2) **5/11+20/9 = 2.68**
3) **Area = 803.84 sq cm , Circumference = 100.48 cm**
4) **30 girls can build a garden in 23 days. How many days will 810 girls build it? = 0.85**
5) **15 oranges costs Rs 33 . How many oranges can you buy for Rs 627? = 285**
6) **9/2 8/7 14/10**
7) **Find the square root of 14 + sqrt(160) = sqrt(10) + sqrt(4)**
8) **(+3a+7b) (-7a-2b) = -55ab -14bb -21aa**
9) **+58ab +7bb -45aa = (-9a-1b) (+5a-7b)**
ANSWERS id=57450 1
==================================
1) **- 14 + 11 - 8 - 2 - 12 + 7 + 3 + 2 = -13**
2) **6/1+18/11 = 7.64**
3) **Area = 1519.76 sq cm , Circumference = 138.16 cm**
4) **6 women can build a bridge in 15 days. How many women will make it in 330 days ? = 0.27**

5) **1 books costs Rs 12 . What is the cost of 9 books? = 108**
6) **1/14 5/4 9/2**
7) **Find the square root of 15 - sqrt(224) = sqrt(8) - sqrt(7), or sqrt(7) - sqrt(8)**
8) **(+7a+5b) (+10a-7b) = +1ab -35bb +70aa**
9) **+50ab -8bb -12aa = (+2a-8b) (-6a+1b)**
ANSWERS id=61526 1
==============================
1) **+ 18 - 14 - 13 + 10 + 18 - 2 + 5 + 10 = 32**
2) **8/3+13/8 = 4.29**
3) **Area = 3215.36 sq cm , Circumference = 200.96 cm**
4) **6 men can build a road in 2 days. How many days will 42 men build it? = 0.29**
5) **44 oranges costs Rs 38 . How many oranges can you buy for Rs 722? = 836**
6) **18/10 13/7 16/8**
7) **Find the square root of 14 - sqrt(192) = sqrt(8) - sqrt(6), or sqrt(6) - sqrt(8)**
8) **(-7a-1b) (-10a+4b) = -18ab -4bb +70aa**
9) **+32ab +24bb -56aa = (-8a+8b) (+7a+3b)**
ANSWERS id=79872 1
==============================
1) **+ 5 - 11 - 6 - 7 + 20 - 15 + 2 - 12 = -24**
2) **4/2+18/17 = 3.06**
3) **Area = 3017.54 sq cm , Circumference = 194.68 cm**
4) **7 men can build a bridge in 5 days. How many men will make it in 90 days ? = 0.39**
5) **40 books costs Rs 47 . What is the cost of 800 books? = 940**
6) **10/2 17/12 15/20**
7) **Find the square root of 12 + sqrt(144) = sqrt(6) + sqrt(6)**
8) **(-7a+8b) (+3a-1b) = +31ab -8bb -21aa**
9) **+32ab +16bb +16aa = (-8a-8b) (-2a-2b)**
ANSWERS id=8237 1
==============================
1) **+ 11 - 4 - 15 - 20 + 9 - 11 + 12 + 3 = -15**
2) **4/19+17/10 = 1.91**
3) **Area = 2826 sq cm , Circumference = 188.4 cm**
4) **11 girls can build a house in 6 days. How many girls will make it in 156 days ? = 0.42**
5) **38 apples costs Rs 30 . What is the cost of 418 apples? = 330**
6) **11/19 8/13 15/14**
7) **Find the square root of 8 - sqrt(60) = sqrt(5) - sqrt(3), or sqrt(3) - sqrt(5)**
8) **(-3a+7b) (+2a-8b) = +38ab -56bb -6aa**
9) **-16ab -3bb +12aa = (+2a-3b) (+6a+1b)**
ANSWERS id=93850 1
==============================
1) **+ 7 - 16 - 3 + 16 - 4 + 18 - 10 + 18 = 26**
2) **17/4+3/16 = 4.44**
3) **Area = 0 sq cm , Circumference = 0 cm**
4) **24 men can build a road in 2 days. How many men will make it in 16 days ? = 3**
5) **23 mangoes costs Rs 40 . What is the cost of 184 mangoes? = 320**
6) **11/6 17/18 9/12**
7) **Find the square root of 8 - sqrt(64) = sqrt(4) - sqrt(4), or sqrt(4) - sqrt(4)**
8) **(-7a-7b) (-7a-9b) = +112ab +63bb +49aa**
9) **-50ab -24bb -25aa = (+5a+4b) (-5a-6b)**
