# Supplementary material for: Sequence/structural analysis of xylem proteome emphasizes pathogenesis-related proteins, chitinases and β-1, 3-glucanases as key players in grapevine defense against Xylella fastidiosa
Source: PeerJ. 2016 May 24;4:e2007. doi: 10.7717/peerj.2007 (PMC4888286; doi:10.7717/peerj.2007)
Supplement: File S3 [file peerj-04-2007-s005.zip › perl_scripts/QP/BACK.1/top.html]

Question bank
